# *Jiangshui*-derived lactic acid bacteria with high hypoglycemic potential: functional properties and fermentation performance of *Sonchus oleraceus Jiangshui*

**DOI:** 10.3389/fmicb.2026.1800013

**Published:** 2026-03-20

**Authors:** Xian-Gang Meng, Hui Yao, Qing-kai Jin, Tuan-Jie Che, Mohamed S. Sheteiwy, Afrah E. Mohammed, Modhi O. Alotaibi, Si-Jing Chang, Yan-Wen Gui

**Affiliations:** 1 Department of Bioengineering, School of Biological and Pharmaceutical Engineering, Lanzhou Jiaotong University, Lanzhou, China; 2 Innovation Center of Functional Genomics and Molecular Diagnostics Technology of Gansu Province, Engineering Laboratory for Biochips of Gansu Province, Lanzhou, China; 3 Department of Integrative Agriculture, College of Agriculture and Veterinary Medicine, United Arab Emirates University, Abu Dhabi, United Arab Emirates; 4 Department of Biology, College of Science, Princess Nourah Bint Abdulrahman University, Riyadh, Saudi Arabia; 5 Microbiology and Immunology Unit, Natural and Health Sciences Research Center, Princess Nourah Bint Abdulrahman University, Riyadh, Saudi Arabia

**Keywords:** lactic acid bacteria, fermentation, functional properties, *Sonchus oleraceus Jiangshui*, hypoglycemic activity, antibacterial properties

## Abstract

This study investigated the probiotic properties of *Lactobacillus paracasei* (LAB 815) isolated from Gansu *Jiangshui* and *Saccharomyces cerevisiae* (SC 8(3)), along with their functional activities and fermentation performances in *Sonchus oleraceus* L. (*S. oleraceus*) *Jiangshui*. Both LAB (815) and SC 8(3) had good gastrointestinal tolerance and auto-aggregation ability, and neither strain showed hemolytic activity. The functional activity assays showed that the fermented *S. oleraceus Jiangshui* (F2) produced through co-fermentation with LAB 815 and SC 8(3) exhibited superior biological activities, including *α*-glucosidase and α-amylase inhibitory effects, ACE inhibitory activity, strong antioxidant capacity, and significant antimicrobial activity against pathogenic bacteria. The results of simulated gastrointestinal fluid test showed that the inhibition rates of *α*-glucosidase, α-amylase and ACE decreased in all groups (F1, F2, F3 and F4), indicating a partial loss of these enzyme inhibitory activities after gastrointestinal transit. However, the F2 group maintained relatively higher activity compared to the other groups and maintained inhibition rates of approximately 50%. The fermentation performance results showed that the F2 produced acid more rapidly and achieved a greater pH reduction compared to other groups (F1, F3, F4). Metabolite analysis showed that F2 resulted in a significant increase in the major metabolites such as amino acids (+339.954 mg/100 mL), alcohols (+29.118%), esters (+25.295%) and organic acids (+15.12%). The sensory evaluation results showed that the overall acceptability of the *S. oleraceus Jiangshui* was significantly improved. The present findings could confirm the key role of co-fermentation of LAB 815 and SC 8(3) in improving the taste quality and potential health functions of *S. oleraceus Jiangshui*.

## 1 Introduction

Fermentation is a traditional and widely practical method for preserving vegetables, with fermented vegetables being a common part of diets in many cultures around the world (Yongsawas et al., 2022; Galena et al., 2024). As people ‘s awareness of the health benefits of fermented vegetables continues to increase, the consumption of fermented vegetables has also increased significantly in recent decades (Liang et al., 2022). Notably, several *in vitro* and *in vivo* studies have reported on the health-promoting properties of fermented vegetables, including antibacterial activity, modulation of intestinal microbiota, anti-cancer effects and anti-diabetic effects, as well as immune regulation (Tan et al., 2024). These functions are believed to be mediated by bioactive metabolites and probiotic effects (Caffrey et al., 2024).

Lactic acid bacteria (LAB), widely present in fermented foods, are recognized as representative probiotics (Jin et al., 2024). A recent study demonstrates that LAB confer systemic health benefits, including therapeutic effects against metabolic disorders such as diabetes and obesity, as well as diseases affecting the cardiovascular, respiratory, central nervous, and digestive systems (Chakravarty et al., 2025). Recent studies highlight the exceptional functional properties of *Jiangshui*-originated LAB strains. For example, *Lactobacillus* from *Jiangshui* sources has been reported to have excellent antioxidant activity (Hu et al., 2023). Consistently, Wu et al. (2021) found that fermented JL-3 from *Jiangshui* can reduce hyperuricemia. Moreover, Khan et al. (2023) found that the fermentation with LAB from *Jiangshui* has significantly improved the nutritional and bioactive substance composition of fermented foods. Jin et al. (2024) reported that LAB isolated from *Jiangshui* exhibit excellent gastrointestinal tolerance, strong antioxidant activity, and cholesterol-lowering effects. Additionally, these strains were also found to enhance both the functional properties and sensory quality of pear juice through fermentation. Therefore, the development of *Jiangshui*-derived LAB as functional starter cultures holds great potential for applications in functional food production.

*Jiangshui* is a traditional fermented food in Northwest China (Gansu), typically made from a variety of vegetables such as celery, cabbage and wild vegetables (Li et al., 2017). LAB and yeast constitute the dominant probiotic microbiota involved in the traditional fermentation process of Gansu *Jiangshui* (Chen et al., 2015). LAB enhance food safety by employing various antibacterial mechanisms including the production of organic acids (such as lactic acid and acetic acid) and bacteriocins (Zhang et al., 2020). At the same time, they can improve sensory characteristics, promote biotransformation and the production of volatile aromatic compounds, and endow fermented foods with unique flavor (Koyuncu and Cabaroglu, 2024). The unique flavor and functional components of fermented foods are typically the result of the metabolic activity and interactions of diverse microbial communities (Cheng et al., 2025). *Jiangshui* also offers a wide range of nutritional and physiological functions, including appetite stimulation, enhanced digestion, reduced risk of heatstroke, cholesterol-lowering effects, and blood pressure regulation (Chen et al., 2015; Wu et al., 2021). Therefore, integrating health-promoting attributes into the development of fermented foods is both necessary and valuable for advancing their functional potential.

*Sonchus oleraceus* (Asteraceae) is a wild edible medicinal plant traditionally used in the treatment of various diseases (Vilela et al., 2009). Its diverse pharmacological properties are attributed to its rich phytochemical composition, which includes flavonoids, flavanols, proanthocyanidins, total phenols, saponins, phytates and alkaloids (Jimoh et al., 2011). Recent studies have demonstrated that *S. oleraceus* possesses a wide range of bioactivities, including antioxidants and anti-diabetes (Teugwa et al., 2013), as well as anti-inflammatory, anti-injury, anti-anxiety effects (Couto et al., 2011). It also exhibits cytotoxic, antibacterial (Xia et al., 2011), and anti-ulcerative colitis activities (Alothman et al., 2018). However, its short shelf life limits its application, as nutrient degradation can occur rapidly during storage. Nonetheless, the probiotic fermentation can prolong the shelf life of processed foods, improve the flavor of products, and increase biological activity (Guo et al., 2023). Consequently, the development of *S. oleraceus*-based fermented *Jiangshui* presents a novel functional food development strategy leveraging microbial-metabolite interactions.

At present, there is a significant gap in research regarding the biological activity and functional characteristics of *S. oleraceus* fermentation. To address this, the present study aimed to: (i) evaluate the probiotic properties of the *Jiangshui*-derived Lactobacillus strain and assess the functional activity and sensory qualities of *S. oleraceus Jiangshui* fermented with a mixed bacterial culture; (ii) investigate the metabolic changes occurring during the fermentation process; and (iii) provide a theoretical basis for the development of novel functional fermented foods.

## 2 Materials and methods

### 2.1 Strains and reagents

In this study, two bacterial strains were selected as research subjects. LAB 815 was isolated from Gansu *Jiangshui* and identified as *Lactobacillus paracasei* based on 16S rRNA gene sequence analysis (99.8% similarity to strain PV104176). The yeast SC 8(3) was isolated from active dry yeast (Angel Yeast Co., Ltd.) and selected as the aroma-producing strain through experimental screening. ITS sequence analysis showed 100% similarity to *Saccharomyces cerevisiae* strain KF633186, confirming it as *Saccharomyces cerevisiae.* Additionally, pathogenic strains including *Staphylococcus aureus*, *Escherichia coli*, and *Klebsiella pneumoniae* were obtained from the Fermentation Engineering Laboratory of Lanzhou Jiaotong University, Gansu Province.

*Sonchus oleraceus* was purchased from Lanzhou (Gansu, China). MRS liquid and agar medium and LB broth were purchased from Solarbio Co., Ltd. (Shanghai, China). Pepsin and trypsin were purchased from Macklin Biochemical Technology Co., Ltd. (Shanghai, China). PNPG, *α*-glucosidase, α-amylase were purchased from Solarbio Co., Ltd. (Beijing, China). ACE kit was purchased from Zike Biotechnology Co., Ltd. (Shenzhen, China). Bile salts, DPPH and pyrogallol were purchased from Biosharp Life Sciences Biotechnology Co., Ltd. (Anhui, China). 3,5-dinitrosalicylic acid (DNS) was purchased from Youpeng Chemical Co., Ltd. (Shanghai, China). Anthrone, xylene, salicylic acid and other reagents were purchased from Xinke Laboratory Equipment Co., Ltd. (Jinan, China).

### 2.2 Functional strains resistant to acid and bile salt

Acid resistance was determined according to the method of Liong and Shah (2005). LAB were cultured in 10 m L MRS medium at 37 °C for 18 h, and yeasts were cultured in 10 mL YPD medium at 25 °C for 96 h. Then, 1 mL of lactic acid bacteria suspension and 1 mL of yeasts were suspended in (pH 2) MRS broth and YPD medium, respectively, and incubated at 37 °C for 3 h. Then the number of living cells (N1) was measured, and the survival rate was determined by comparing N1 with N0.

The bile salt tolerance test was performed according to the method of Trivedi et al. (2013). The overnight cultures of each strain were inoculated 10% (v/v) into MRS broth and YPD medium (containing 0.3% bovine bile (w/v)) and incubated at 37 °C for 3 h. Calculate the survival percentage as shown in Equation 1:


Survival rate(%)=N1/N0×100 (1)

Where, N1 represents the number of viable cells after incubation; N0 represents the initial number of cells (logCFU/mL).

### 2.3 Hydrophobicity and auto-aggregation

The surface hydrophobicity of LAB was determined according to the method described by Sadishkumar and Jeevaratnam (2017). with slight modification. The LAB strains were cultured in MRS broth at 37 °C for 18 h, while the yeast strains were grown in YPD broth at 25 °C for 96 h. Following incubation, the cells were harvested by centrifugation at 6000 × g for 15 min. Each strain was washed twice with sterile phosphate buffered saline (PBS, 0.1 M, pH 7.4) containing 0.80% NaCl, 0.02% KCl, 0.02% KH_2_PO_4_ and 0.22% disodium hydrogen phosphate, and suspended in 0.1 M KNO_3_ to an optical density (OD_600_) of 0.5 ± 0.05 (At). A round 1.0 mL xylene was added to 3.0 mL bacterial suspension. The mixture was pre-incubated at 37 °C for 10 min, vortexed for 2 min, and then incubated at 37 °C for an additional 20 min. The two phases (water and xylene) were separated. The aqueous phase was collected and its OD_600_ (A0) was determined. Hydrophobicity was calculated as shown in Equation 2:


Hydrophobicity(%)=(1−At/A0)×100 (2)

Where, At and A0 are the absorbance values of the suspension before and after the reaction.

Auto-aggregation determination was performed according to the method of Wang et al. (2021) and minor modifications were made. The bacterial cells were harvested at 4000 g for 10 min, washed twice with PBS, and the OD_600_ of the bacteria was adjusted to 0.5 ± 0.05 with sterile water, recorded as A0. Then they were incubated at 37 °C for 3 h. The auto-aggregation ability of absorbance measured at OD600 of the upper liquid is calculated as shown in Equation 3:


Auto−aggregation(%)=(1−At/A0)×100 (3)

Where, At represents the absorbance of 3 h; A0 represents the absorbance of 0 h.

### 2.4 Hemolytic activity

The hemolytic activity of LAB 815 and SC 8(3) was evaluated according to the method described by Pieniz et al. (2014) with slight modifications, using the spot inoculation method. Activated cultures of the strains were adjusted to an OD₆₀₀ of 0.5, and 10 μL of each suspension was spotted onto Columbia blood agar plates (5% sheep blood). The plates were incubated at 37 °C for 48 h. *Staphylococcus aureus* ATCC 6538 and *Escherichia coli* DH5*α* served as positive and negative controls, respectively. Hemolytic activity was classified as α-hemolysis (green zone), *β*-hemolysis (clear zone), or *γ*-hemolysis (no zone).

### 2.5 Preparation and fermentation of *Sonchus oleraceus Jiangshui*

Fresh *S. oleraceus* was selected to remove rotten leaves, old leaves, roots, etc. and wash them. A round 40 g was weighed, boiled in 1000 mL boiling water for 1 min, and placed in a fermentation tank. 3 g of flour (The flour was purchased from a local market in Lanzhou City, China.) and 3 g of sucrose were added to 300 mL boiling water. The process was continuously stirred to form a noodle soup. Cooled to 30 °C and placed in a jar, The inoculation ratio (high hypoglycemic LAB: aroma-producing yeast = 1:1) was inoculated into 300 mL noodle soup with 14% (v/v) inoculation amount, and fermented at 30 °C for 3 days. All fermentation parameters were determined based on previous optimization experiments. To evaluate the effect of different microbial combinations on the fermentation performance, four experimental groups (F1–F4) were designed as shown in Table 1. After fermentation, the samples were centrifuged at 10,000 × g for 15 min at 4 °C to remove microbial cells and insoluble particles. The resulting supernatants were collected and immediately used for subsequent analysis.

**Table 1:** Experimental design and microbial composition of fermentation groups (F1–F4).

Group	Microbial composition	Purpose
F1	LAB 815	Single-strain fermentation
F2	LAB 815 + SC 8(3)	Co-fermentation
F3	*Jiangshui Yinzi* (traditional starter)	Traditional fermentation control
F4	Natural fermentation (Sterile water)	Blank control

### 2.6 *In vitro* enzyme inhibitory activity of *Sonchus oleraceus Jiangshui*

(1) Inhibition rate of α-glucosidase

Refer to the method of Dou et al. (2019) with modifications 1 mL of phosphate buffer (pH = 7.0), 0.2 mL of *α*-glucosidase solution, 0.4 mL of *Jiangshui* fermentation broth were added to the test tube and shaken, 37 °C water bath for 10 min, 0.5 mL of 10 g/L PNPG solution was added to shake, then 37 °C water bath for 10 min, and finally 8 mL of 0.1 mol/L Na_2_CO_3_ solution was added. The absorbance value was measured at 405 nm, The α-glucosidase inhibition rate was calculated as shown in Equation 4:


α−glucosidase inhibition rate(%)=(A1−A2)/A3×100 (4)

Where, A1 is the absorbance value of adding α-glucosidase solution, fermented *Jiangshui* and substrate PNPG, A2 is the absorbance value of adding α-glucosidase solution and fermented *Jiangshui*, A3 is the absorbance value of adding α-glucosidase solution and PNPG.

(2) Inhibition rate of α-amylase

Refer to the method of Won et al. (2021), 200 μL fermentation supernatant was mixed with 200 μL 10 g/L soluble starch solution and incubated at 37 ° C for 10 min, then 200 μL α-amylase working solution was added and incubated for 10 min. Subsequently, 400 μL DNS was added, boiled water bath for 5 min, and 4 mL deionized water was added after cooling. After mixing, the OD value at 540 nm was measured in the microplate reader, and the inhibition rate was calculated as shown in Equation 5:


$$\;\alpha -\text{amylase inhibition rate}\;(\%)=[\begin{array}{l} 1-(A3-A4)/\\ \left(A2-A1\right) \end{array}]\times 100\;$$ (5)

Where, A1 is the absorbance of the sample without the sample containing the enzyme; A2 is the absorbance without enzyme and sample; A3 is the absorbance containing the enzyme and the sample; A4 is the absorbance of the sample without enzyme.

(3) Determination of glucose dialysis retardation index (GDRI)

1 mL sample was added to 10 mL 0.1 mol/L glucose solution, and the sample was continuously shaken at 37 °C for 1 h, then transferred to a dialysis bag with a molecular weight cut-off of 7,000. At the same time, blank control sample (with glucose, without sample) and sample control sample (with sample, without glucose) were prepared. The dialysis bag was placed in a 500 mL beaker, oscillated at 37 °C for 60 min, and 2 mL of glucose dialysate was taken every 30 min. The mass concentration of glucose was determined, and the glucose dialysis delay index (GDRI) was calculated as shown in Equation 6 and Zheng et al. (2021):


GDRI(%)=[1−(A1−A2)/A3]×100 (6)

Where, A1 is the glucose concentration of the sample solution; A2 is used as the glucose concentration of the sample control; A3 is the glucose concentration of the blank control. The unit is μg/mL.

### 2.7 ACE inhibitory activity of *Sonchus oleraceus Jiangshui in vitro*

The activity of ACE was detected by ACE kit. The indicator working solution was prepared according to Table 2, and the indicator working solution was added and fully mixed. The absorbance value A1 at 15 s was measured at 340 nm, and the reaction was quickly placed at 37 °C for 5 min. The absorbance value A2 was measured at 5 min and 15 s, and ΔA determination = A1 determination-A2 determination, ΔA blank = A1 blank-A2 blank, ΔA = ΔA determination-ΔA blank. Blank tube only needs to do 1–2 times. and the ACE activity was calculated as shown in Equation 7:


$$\begin{array}{l} \mathrm{ACE}\;\text{activity}\;(U/\mathrm{ML})=\mathrm{\Delta A}/(\varepsilon \times d)\times V_{\text{anti}-\text{total}}\times \\ 10^{9}÷V_{\text{sample}}\times T\times F \end{array}$$ (7)

**Table 2:** Preparation of working solution for the indicator of ACE activity determination.

Reagent name (μL)	Blank tubes	Measurement tubes
Sample supernatant	―	500
Reagent 1	500	―
Reagent 2	500	500

Where, ε is the molar extinction coefficient of FAPGG (N-[3-(2-Furyl) acryloyl]-Phe-Gly-Gly) at 340 nm, its value is 758 L/(mol·cm); d is1mLquartz cuvette light path, its value is 1 cm; Vanti-total is the total volume of the reaction system, its value is 1 xL; V sample is sample volume added in the reaction system, its value is 0.5 mL; T is reaction time, its value is 5 min; F is Dilution multiple.

### 2.8 Tolerance to simulated gastric and intestinal fluid

The method of Cruz-Huerta et al. (2014) was used with modifications to simulate gastrointestinal digestion. 2 mL sample was added to 10 mL simulated gastric juice (1 g pepsin was dissolved in 100 mL deionized water and 1.64 mL 0.1 mol/L HCl was added), and then incubated at 37 °C for 180 min. The reaction was terminated by heating at 80 °C for 5 min, and the *α*-amylase, α-glucosidase inhibitory activity and ACE activity were determined. Also, 2 mL sample was added to 10 mL simulated intestinal fluid (0.68 g K_2_HPO_4_ and 1 g trypsin were dissolved in 100 mL deionized water, and the pH value was adjusted to 6.8 with dilute NaOH solution), and then heated at 37 °C for 180 min. After the reaction was terminated by heating, the α-amylase, α-glucosidase inhibitory activity and ACE activity were determined. All inhibition rates were measured in triplicate.

### 2.9 Antioxidant activity

(1) Hydroxyl radical scavenging activity

According to Zhang et al. (2020) method, small modifications are made to measure. In the determination tube, 1 mL FeSO_4_ (9 mmol/L), 1 mL salicylic acid (9 mmol/L), 2 mL sample were added in turn, and mixed well. In the blank tube, 1 mL FeSO_4_ (9 mmol/L), 1 mL salicylic acid (9 mmol/ L), 2 mL distilled water were added in turn, and mixed well. Then 1 mL H_2_O_2_ (8.8 mmol/L) was added and mixed well. The mixture was reacted at 37 ° C for 30 min, and then detected at 510 nm. The Hydroxyl free radical scavenging rate was calculated as shown in Equation 8:


$$\begin{array}{l} \text{Hydroxyl free radical}\;\\ \text{scavenging rate}\;(\%)=[1-(\mathrm{Ai}-\mathrm{Aj})/A0]\times 100\; \end{array}$$ (8)

Where, A0 is the absorbance of blank control solution; Ai is the absorbance of the sample solution; Aj is the absorbance of the sample itself.

(2) Superoxide anion radical scavenging activity

Superoxide anion radical scavenging activity test was slightly modified according to the method of Yu et al. (2019). 2 mL of sample and 0.4 mL of pyrogallol (10 mmol/L) were added into 4.5 mL of 50 mmol/L Tris–HCl buffer (pH 8.2) for 5 min, and then 1 mL of 8 mmol/L HCl solution was added to end the reaction. The absorbance value was measured at 325 nm, and distilled water was used instead of sample as blank control. The superoxide anion scavenging rate was calculated as shown in Equation 9:


$$\begin{array}{l} \text{Superoxide anion radical}\;\\ \text{scavenging rate}\;(\%)=[1-(\mathrm{Ai}-\mathrm{Aj})/A0]\times 100\; \end{array}$$ (9)

Where, A0 is the absorbance of blank control solution; Ai is the absorbance of the sample solution; Aj is the absorbance of the sample itself.

(3) DPPH (2, 2-diphenyl-1-picrylhydrazyl) radical scavenging activity

The DPPH scavenging ability was determined according to the experimental method of Liu et al. (2011). The 2 mL of sample was mixed with 2 mL of 0.1 mmol/L DPPH ethanol solution, and reacted at room temperature in dark for 30 min. The absorbance value was measured at 517 nm. The DPPH radical scavenging rate was calculated as shown in Equation 10:


$$\begin{array}{l} \text{DPPH radical}\;\\ \text{scavenging rate}\;(\%)=[1-(\mathrm{Ai}-\mathrm{Aj})/A0]\times 100\; \end{array}$$ (10)

Where, A0 is control group absorbance; Ai is sample group absorbance value; Aj is blank group absorbance value.

### 2.10 Antibacterial properties

The antibacterial activity was determined by slightly modifying the pore diffusion method of Nami et al. (2019). *Staphylococcus aureus*, *Escherichia coli* and *Klebsiella pneumoniae* were used as indicator bacteria. The three strains were cultured in a 37 °C incubator for 24 h. The diluted bacterial solution was evenly coated on the surface of LB solid medium, and 80 μL of samples of different fermentation types were injected into the hole after uniform drilling with a puncher. Then it was placed in a 37 °C incubator for 12 h. The diameter of the inhibition zone (mm) was measured with a vernier caliper, and the size of the inhibition zone was used to characterize the antibacterial effect.

### 2.11 Physicochemical property of *Sonchus oleraceus Jiangshui*

The pH, total acid and reducing sugar content were determined during the simulated fermentation experiment of *S. oleraceus Jiangshui*. The pH value in the process of *S. oleraceus Jiangshui* fermentation was detected by pH meter. The content of reducing sugar was determined by 3,5-dinitrosalicylic acid (DNS) method (Zhang et al., 2017). Determination of total acid content was carried out by titrating the total acid at the phenolphthalein endpoint with 0.1 mol/L NaOH using the method of Wu et al. (2012).

### 2.12 Determination of metabolites of *Sonchus oleraceus Jiangshui*

(1) Amino acid

The amino acid content was determined according to Park et al. (2024) with modifications. *Jiangshui* sample was centrifuged at 12,000 × g for 10 min at 4 °C to remove solids, and the supernatant was filtered through a 0.22 μm sterile PVDF membrane (Millipore, USA). The filtrate was analyzed using a JASCO LC-2000 Plus HPLC system (JASCO, Tokyo, Japan) equipped with a fluorescent detector and an AccQ-Tag Amino Acids C18 column (3.9 mm × 150 mm, 4 μm; Waters, USA). The mobile phase consisted of (A) AccQ-Tag/water (10:90, v/v) and (B) acetonitrile/water (60:40, v/v) with the following gradient: 100% A (0 min), 98% A (5 min), 93% A (15 min), 90% A (19 min), 67% A (32–33 min), 0% A (34–37 min), and 100% A (38 min, held for 2 min). The flow rate was 1 mL/min, the column temperature was 37 °C, and the injection volume was 10 μL. Amino acids were derivatized using the AccQ-Fluor Reagent Kit (Waters, USA) and detected at 254 nm by a photodiode array detector (MD-2018 Plus, JASCO). Quantification was based on a five-point calibration curve of an amino acid standard (WAT088122, Waters). All samples were analyzed in duplicate, and the results showed a relative standard deviation of < 5%.

(2) Volatile compounds

Volatile compounds were determined according to the method modified by Sevindik et al. (2022), which were detected by a gas chromatograph (Agilent 7890A, USA) with a mass selective detector (MSD) (Agilent 5975C, USA). Samples (5 mL) and NaCl (2 g) were added to headspace bottles. A HP-INNOWAX capillary column (0.25 mm × 30 m ID, 0.25 μm film thickness) was selected to separate volatile compounds. After holding at 60 °C for 3 min, the column temperature was increased to 230 °C at a rate of 2 °C/min and then increased to 245 °C at a rate of 3 °C/min and kept at 245 °C for 20 min. Helium was used as a carrier at a flow rate of 1 mL/min. The volatile compounds were identified by mass spectrometry in comparison of the NIST10 database. The concentration of volatile compounds was determined by using an in-ternal standard (2-octanol).

### 2.13 Sensory evaluation

The sensory characteristics of fermented *S. oleraceus Jiangshui* were evaluated by trained sensory testing experts (5 males and 5 females, aged 24–28 years) from the School of Biology and Pharmaceutical Sciences, Lanzhou Jiaotong University, using a method based on Jin et al. (2024) with slight modifications. The written informed consent of all experts was obtained before the test. Sensory evaluation was carried out in blind study. In short, four kinds of fermented *S. oleraceus Jiangshui* (F1, F2, F3, F4) were placed in a clear odorless cup, marked with three-digit codes, and randomly presented to the team members to evaluate sensory attributes, including color, aroma, taste, and turbidity. The tester rinses the mouth with water before and during the test. A 4-point scale (0–4) was used, and the final score was calculated as the average of scores from 10 panelists.

### 2.14 Data statistics and analysis

All experiments were performed in triplicate, and the results are presented as mean ± standard deviation (SD). Statistical analyses were conducted using IBM SPSS Statistics (version 27.0; IBM Corp., Armonk, NY, USA), with one-way analysis of variance (ANOVA) followed by *post hoc* tests (Tukey’s test) to assess significant differences among groups. A *p*-value < 0.05 was considered statistically significant. GraphPad Prism (version 9.5.0; GraphPad Software, San Diego, CA, USA) and OriginPro (version 9.1; OriginLab Corporation, Northampton, MA, USA) were used to generate data visualizations and graphical representations.

## 3 Results

### 3.1 Probiotic properties of LAB 815 and SC 8(3)

#### 3.1.1 Acid and bile salt tolerance

The survival rates of LAB 815 and SC 8 (3) in simulated gastric juice environment were 84.15 and 80.21%, respectively, both exceeding 80%, indicating strong tolerance to acidic gastric conditions. Under simulated intestinal juice conditions, the survival rates were 78.24 and 76.25%, respectively (Table 3). These results suggest that both strains are capable of withstanding harsh gastrointestinal environments and may effectively exert their probiotic functions.

**Table 3:** The characteristics of probiotics (acid resistance, bile salt resistance, hydrophobicity and self-aggregation ability) of LAB 815 and SC 8(3) were determined.

Strains	Survival in gastric fluid (%)	Survival in intestinal fluid (%)	Hydrophobicity (%)	Auto-aggregation (%)
LAB 815	84.15 ± 4.56	78.24 ± 2.34	34.03 ± 1.56	27.24 ± 2.11
SC 8(3)	80.21 ± 3.73	76.25 ± 6.54	36.85 ± 3.25	20.88 ± 1.52

All values in the table were mean ± standard deviation (*n* = 3).

#### 3.1.2 Hydrophobicity and auto-aggregation

The hydrophobicity (xylene) of LAB 815 and SC 8(3) were 34.03 and 36.85%, respectively, and the auto-aggregation rates of LAB 815 and SC 8(3) were 27.24 and 20.88%, respectively (Table 3). These results indicate that both LAB 815 and yeast SC 8(3) possess moderate hydrophobicity and auto-aggregation ability, suggesting a strong potential for adhesion to intestinal epithelial cells.

#### 3.1.3 Hemolytic activity

**Figure 1:**
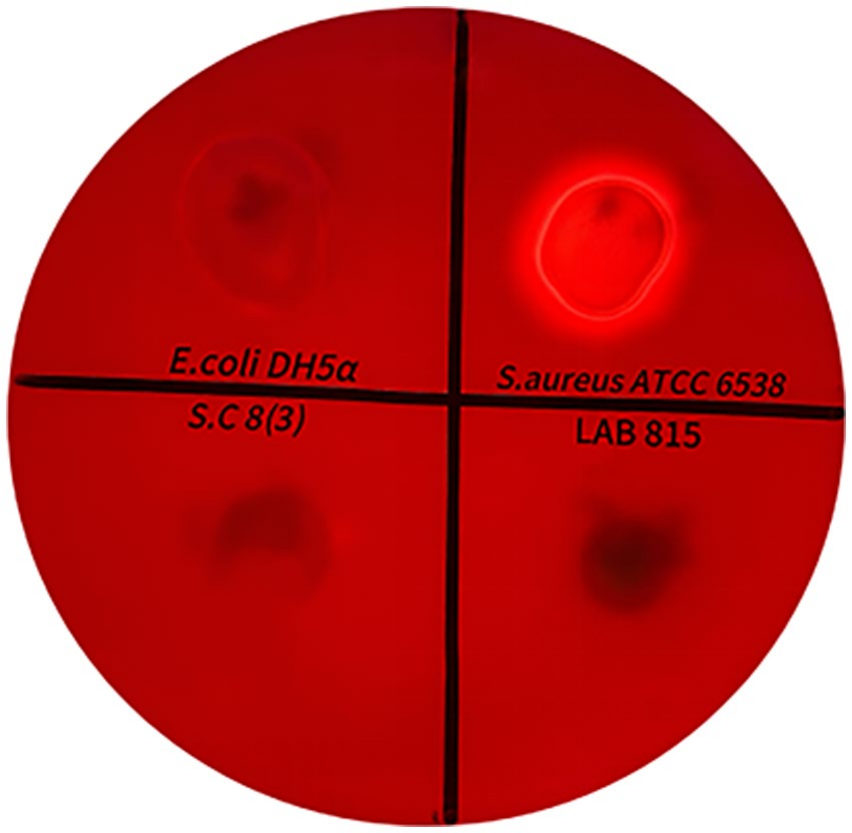
Hemolytic activity of LAB 815 and SC 8(3) on sheep blood agar plates. *Escherichia coli* DH5a and *Staphylococcus aureus* ATCC 6538 were used as negative and positive controls, respectively.

The hemolytic activity of LAB 815 and SC 8(3) was assessed on Columbia blood agar plates. As shown in Figure 1, *Staphylococcus aureus* ATCC6538 (positive control) produced a clear transparent zone around colonies, indicating *β*-hemolysis, while *Escherichia coli* DH5*α* (negative control) showed no hemolytic zone (*γ*-hemolysis). Under the same conditions, both LAB 815 and SC 8(3) exhibited no hemolytic zones around the colonies, corresponding to γ-hemolysis. These results indicate that both strains are non-hemolytic.

### 3.2 Functional properties of *Sonchus oleraceus Jiangshui*

#### 3.2.1 *α*-Glucosidase, α-amylase inhibitory activity and GDRI

**Figure 2:**
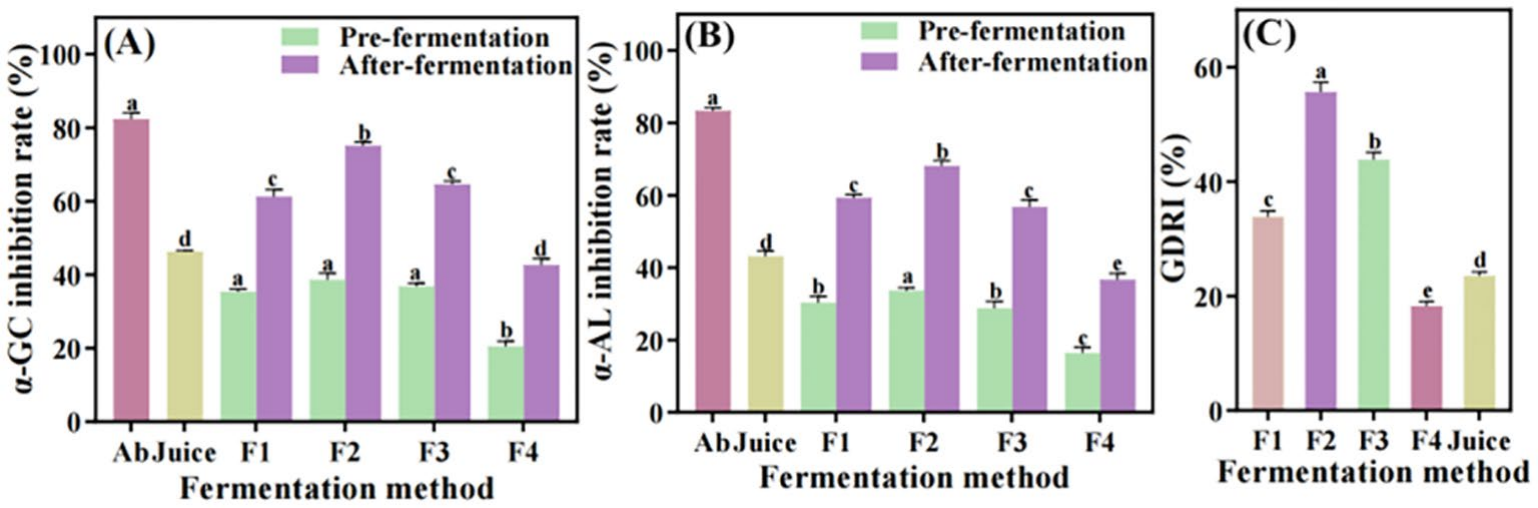
Determination of hypoglycemic function of different fermentation methods of *S. oleraceus Jiangshui* and unfermented *S. oleraceus* juice (Juice). *α*-GC inhibition rate is α-glucosidase inhibition rate; α-AL inhibition rate is α-amylase inhibition rate; GDRI is glucose dialysis retardation index; F1 is LAB 815 fermented *S. oleraceus Jiangshui*; F2 is LAB 815 and SC 8(3) mixed fermented *S. oleraceus Jiangshui*: F3 is traditional starter culture (*Jiangshui Yinzi*) fermented *S. oleraceus Jiangshui*; F4 is natural fermented *S. oleraceus Jiangshui* (CK). AB is acarbose (positive control): Juice is fresh *S. oleraceus* juice. All values in the figure were mean standard deviation (*n* = 3). There were significant differences in the values of different lowercase letters (*p* < 0.05, one-way ANOVA followed by Tukey’s test). **(A)** α-glucosidase inhibition rate; **(B)** α-amylase inhibition rate; **(C)** Glucose dialysis retardation index (GDRI).

Inhibition of α-glucosidase and α-amylase activities is a key mechanism for regulating postprandial blood glucose levels. In our study, four types of *S. oleraceus Jiangshui* and *S. oleraceus* juice demonstrated inhibitory effects on both enzymes. Specifically, *S. oleraceus* juice inhibited α-glucosidase and α-amylase activities by 46.19 and 43.11%, respectively (Figures 2A,B). These findings suggest that *S. oleraceus* juice has the potential to contribute to blood glucose regulation through enzymatic inhibition. However, after the introduction of probiotic fermentation, the inhibition rates of α-glucosidase and α-amylase in F1, F2 and F3 were significantly increased (*p* < 0.001). Among them, formulation F2 exhibited the highest inhibitory activity, with α-glucosidase and α-amylase inhibition rates of 75.14 and 68.14%, respectively. These values were significantly higher than those observed in F1 and F3 (*p* < 0.001), which were 91.27 and 81.77% of the inhibition levels achieved by acarbose, respectively. The inhibition rate of α-amylase in F1 (59.3%) was higher than that in F3 (56.8%), demonstrating a 2.5% increase. While the inhibition rate of α-glucosidase in F1 (61.26%) was lower than that in F3 (64.6%), showing a 3.34% reduction. But the inhibition rate of α-glucosidase and α-amylase in F4 were lower than that of *S. oleraceus* juice. The results showed that the inhibition of *α*-amylase and α-glucosidase activity was significantly enhanced after fermentation of *S. oleraceus* (Figures 2A,B). Results showed that the GDRI values of F1, F2, and F3 after probiotic fermentation were significantly higher than that of *S. oleraceus* juice (*p* < 0.001), with F1 and F3 reaching 33.86 and 43.84%, respectively. The GDRI of F2 exhibited the highest GDRI (55.67%), which was 2.36-fold higher than that of *S. oleraceus* juice. While the GDRI of F4 was 5.31% lower than that of *S. oleraceus* juice. The results showed that the GDRI of *S. oleraceus* was significantly increased after fermentation by probiotics (Figure 2C).

#### 3.2.2 ACE inhibitory activity

**Figure 3:**
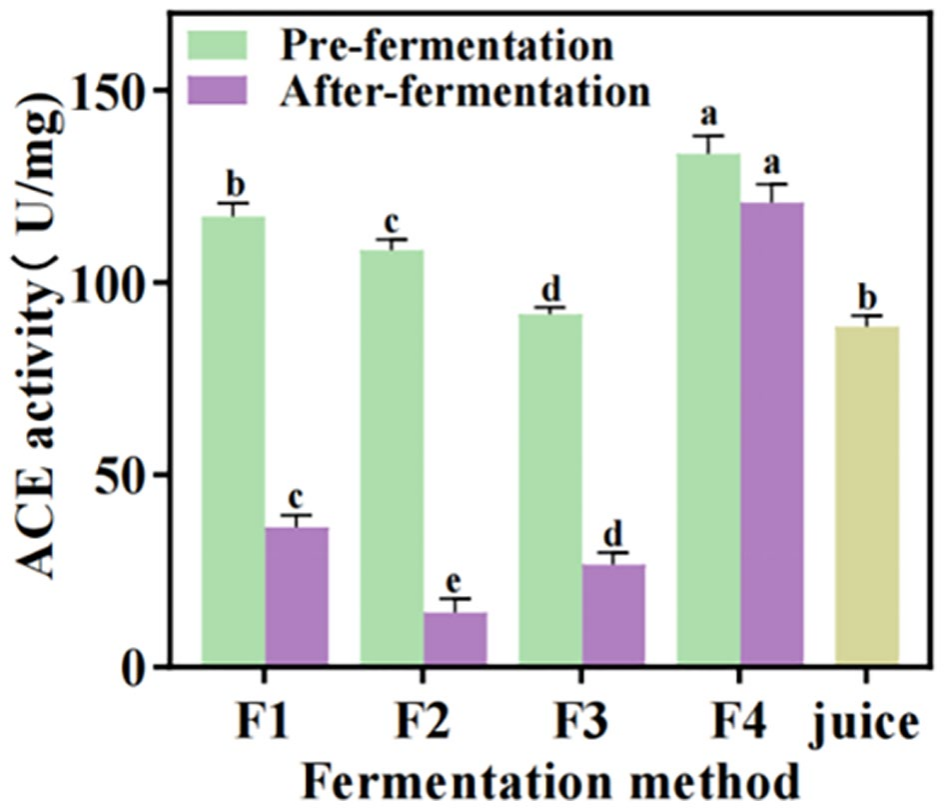
Determination of ACE activity in different fermentation methods of *S. oleraceus Jiangshui* and unfermented *S. oleraceus* juice (Juice). F1 is LAB 815 fermented *S. oleraceus Jiangshui*; F2 is LAB 815 and SC 8(3) mixed fermented *S. oleraceus Jiangshui*; F3 is traditional starter culture (*Jiangshui Yinzi*) fermented *S. oleraceus Jiangshui*: F4 is natural fermented *S. oleraceus Jiangshui* (CK). Juice is fresh *S. oleraceus* juice. All values in the figure were mean ± standard deviation (*n* = 3). There were significant differences in the values of different lowercase letters (*p* < 0.05, one-way ANOVA followed by Tukey’s test).

*In vitro* ACE inhibitory activity of the fermented *S. oleraceus Jiangshui* was measured, and a significant reduction in ACE activity was observed following probiotic fermentation (Figure 3). The ACE activity in F2 exhibiting the most pronounced decrease (94.2361 U/mg), its ACE inhibition rate was the highest (86.86%), followed by F3 and F1, reached 71 and 69.8%, respectively. In contrast, the ACE activity in F4 showed only a marginal decline (12.7437 U/mg), the ACE inhibition rate was 9.5%. Notably, unfermented *S. oleraceus* juice showed an ACE activity of 88.4958 U/mg, confirming that probiotic fermentation effectively reduced ACE activity (Figure 3). These results suggest that probiotic fermentation enhances the ACE inhibitory activity of *S. oleraceus Jiangshui*.

#### 3.2.3 Gastrointestinal fluid simulation

**Figure 4:**
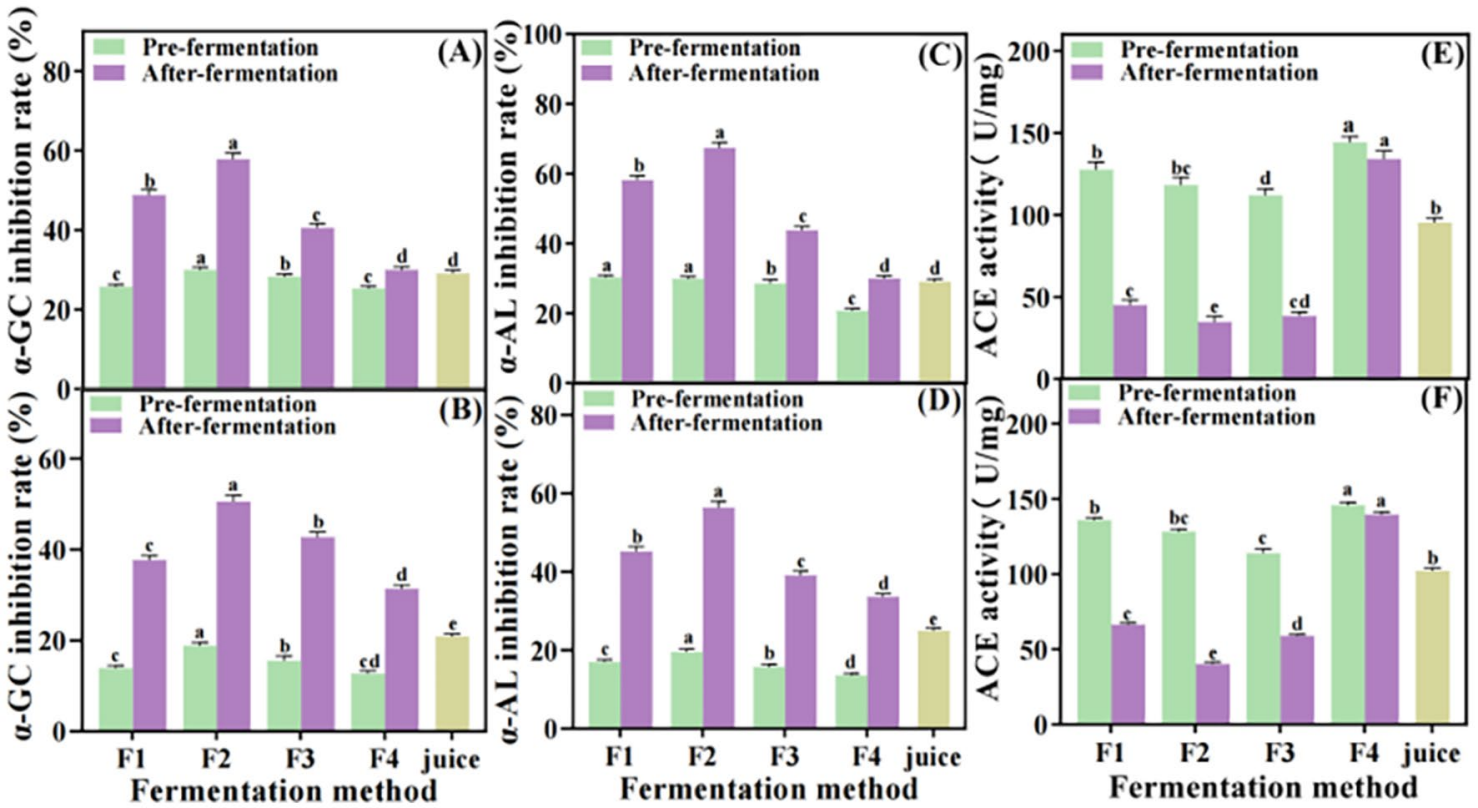
The inhibition rates of α-glucosidase **(A)**, α-amylase **(C)**, and ACE activity **(E)** *in vitro* gastric juice simulation and α-glucosidase **(B)**, α-amylase **(D)**, and ACE activity **(F)** in intestinal fluid simulation of *S. oleraceus Jiangshui* and unfermented *S. oleraceus* juice (Juice) with different fermentation methods were studied. α-GC inhibition rate is α-glucosidase inhibition rate; α-AL inhibition rate is α-amylase inhibition rate; F1 is LAB 815 fermented *S. oleraceus Jiangshui*; F2 is LAB 815 and SC 8(3) mixed fermented *S. oleraceus Jiangshui*; F3 is traditional starter culture (*Jiangshui Yinzi*) fermented *S. oleraceus Jiangshui*; F4 is natural fermented *S. oleraceus Jiangshui* (CK); Juice is fresh *S. oleraceus* juice. All values in the figure were mean standard deviation (*n* = 3). There were significant differences in the values of different lowercase letters (*p* < 0.05, one-way ANOVA followed by Tukey’s test).

The ability of *S. oleraceus Jiangshui* fermented by different fermentation methods was evaluated in the present study to reduce blood glucose and blood pressure in simulated gastrointestinal fluid *in vitro*. The rate of *α*-glucosidase inhibition, α-amylase inhibition as well as ACE activity have changed *in vitro* simulated gastrointestinal fluid experiments. The inhibition rates of α-glucosidase and α-amylase were decreased after exposure to simulated gastric juice for 180 min. However, the inhibition rates of α-glucosidase and α-amylase in F1, F2, F3 and F4 after fermentation were higher than those before fermentation (Figures 4A,C). The inhibition rates of α-glucosidase and α-amylase in F2 were 57.84 and 67.39% respectively, which were 28.73 and 38.4% higher than those of unfermented *S. oleraceus* juice, respectively. The inhibition rates of α-glucosidase and α-amylase in F1 were 48.85 and 58.13%, respectively, while the inhibition rates in F3 were 40.59 and 43.78%, respectively. The inhibition rates of α-glucosidase and α-amylase in F2 were significantly higher than those in F1 and F3 (*p* < 0.001). The results showed that there was no significant difference in the inhibition rates of α-glucosidase and α-amylase between F4 and *S. oleraceus* juice (Figures 4A,C). The ACE activity increased from 14.2479 (U/mg) to 34.6648 (U/mg) in F2 under gastric juice simulation, it means that the ACE inhibition rate decreased from 86.86 to 70.7% (Figure 4E). After exposure to simulated intestinal fluid for 180 min, the inhibition rates of α-glucosidase and α-amylase in F2 decreased to 50.52 and 56.37%, respectively. However, the inhibition rates of α-glucosidase and α-amylase were significantly increased in all fermented samples compared to their unfermented samples (*p* < 0.001). Among them, sample F2 exhibited the most pronounced effect, which were 29.64 and 31.45% higher than those of unfermented *S. oleraceus* juice, respectively. In contrast, the inhibition rates of α-glucosidase and α-amylase in *S. oleraceus* juice were significantly lower than those of F1, F2, F3 and F4 under intestinal fluid simulation (*p* < 0.001, Figures 4B,D). The ACE activity of F1, F2, F3 and F4 was increased to 66.4958 (U/mg), 40.0817 (U/mg), 58.9127 (U/mg) and 139.5607 (U/mg), respectively and the ACE inhibition rate of F1, F2, F3 and F4 were 51.07, 68.72, 48.28, and 4.39%, respectively. The inhibition rate of ACE in F2 was the highest (Figure 4F).

#### 3.2.4 Antioxidant activity

**Figure 5:**
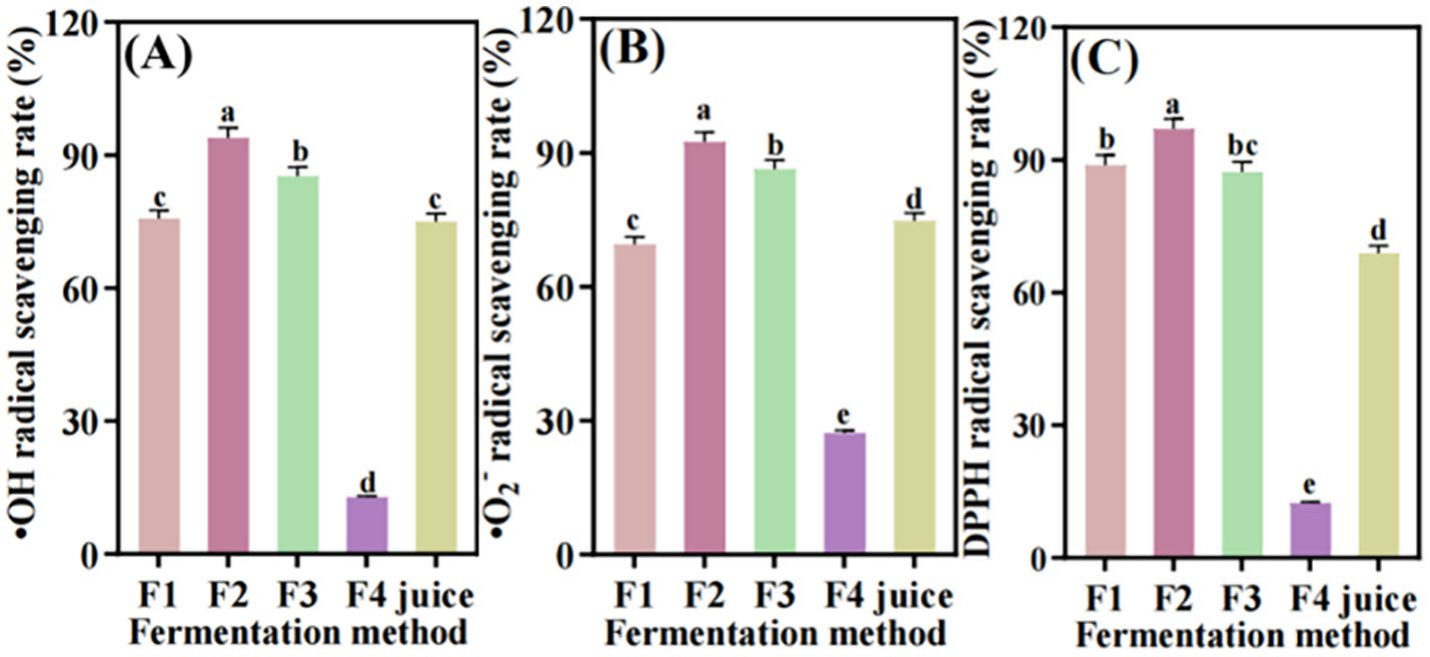
Determination of the scavenging ability of hydroxyl radical **(A)**, superoxide anion radical **(B)**, and DPPH free radical **(C)** in *S. oleraceus Jiangshui* and unfermented *S. oleraceus* juice (Juice) with different fermentation methods. OH, Hydroxyl radical scavenging rate; O_2_, Superoxide anion scavenging rate: DPPH: DPPH free radical scavenging rate; F1 is LAB 815 fermented *S. oleraceus Jiangshui*; F2 is LAB 815 and SC 8(3) mixed fermented *S. oleraceus Jiangshui*; F3 is traditional starter culture (*Jiangshui Yinzi*) fermented *S. oleraceus Jiangshui*; F4 is natural fermented *S. oleraceus Jiangshui* (CK); Juice is fresh *S. oleraceus* juice; All values in the figure were mean standard deviation (*n* = 3). There were significant differences in the values of different lowercase letters (*p* < 0.05, one-way ANOVA followed by Tukey’s test).

The scavenging activities of hydroxyl radical, superoxide anion radical and DPPH free radical of *S. oleraceus Jiangshui* were evaluated using different fermentation methods. Four different fermentation types of *S. oleraceus Jiangshui* and *S. oleraceus* juice were detected and all have certain ability to scavenge hydroxyl radical. Results stated that F2 showed excellent hydroxyl radical scavenging ability, which was 93.9%. The hydroxyl radical scavenging rates of F1 (75.7%) and F3 (85.2%) were significantly higher than that of F4 (12.8%) (*p* < 0.001, Figure 5A). There were no significant differences observed between F1 and unfermented *S. oleraceus* juice (Figure 5A). Different fermentation types showed different superoxide anion scavenging activity. Among the samples, F2 exhibited the highest superoxide anion scavenging rate at 92.4%, followed by F3 (86.3%), *S. oleraceus* juice (74.7%), F1 (69.5%), and F4 (27.2%) (Figure 5B). The primary DPPH free radical scavenging activity was observed in F1, F2, and F3, which were fermented with the introduction of probiotics. The scavenging rate of DPPH free radical in F2 was 97%, which was significantly higher than that in F4 (12.4%, *p* < 0.001) and unfermented *S. oleraceus* juice (68.9%, *p* < 0.001), while there was no significant difference between F1 and F3 (Figure 5C). These results indicated that *S. oleraceus Jiangshui* and juice of *S. oleraceus* have certain antioxidant activity, and F2 showed the most excellent hydroxyl, superoxide anion, DPPH free radical scavenging activity.

#### 3.2.5 Antimicrobial activity

The inhibitory effects of four different fermentation types of *S. oleraceus Jiangshui* on *Staphylococcus aureus*, *Escherichia coli* and *Klebsiella pneumoniae* were determined as shown in Table 4. In this study, all four types of *S. oleraceus Jiangshui* exhibited inhibitory activity against *Staphylococcus aureus*, *Escherichia coli*, and *Klebsiella pneumoniae*. Among them, F2 demonstrated the largest inhibition zones against all three pathogens, closely approaching those produced by kanamycin. The inhibition zone of F2 against *Staphylococcus aureus*, *Escherichia coli* and *Klebsiella pneumoniae* was 12.2 mm, 9.5 mm and 10.8 mm, respectively, which reached 60.7, 60.9 and 59.67% of the inhibition zone of kanamycin, respectively. However, the inhibitory zone of F4 against the three pathogens was significantly smaller than those of F1, F2 and F3 (*p* < 0.001), while there was no significant difference observed in the inhibition zone of F1, F2 and F3 against *Escherichia coli* (*p* < 0.001).

**Table 4:** The inhibitory effect of inoculated fermentation and natural fermentation of *S. oleraceus Jiangshui* on three pathogenic bacteria (*Staphylococcus aureus*, *Escherichia coli*, *Klebsiella pneumoniae*) was determined.

Pathogenic bacteria	Fermentation method (mm)				Kanamycin
	F1	F2	F3	F4	
*Staphylococcus aureus*	10.6 ± 0.34^c^	12.2 ± 0.13^a^	11.7 ± 0.16^b^	5.4 ± 0.21^d^	20.1 ± 0.23
*Escherichia coli*	9.1 ± 0.27^b^	9.5 ± 0.32^a^	9.1 ± 0.43^b^	4.0 ± 0.24^c^	15.6 ± 0.33
*Klebsiella pneumoniae*	10.1 ± 0.29^b^	10.8 ± 0.15^a^	9.1 ± 0.36^c^	4.3 ± 0.35^d^	18.1 ± 0.15

F1 is LAB 815 fermented *S. oleraceus Jiangshui*; F2 is LAB 815 and SC 8(3) mixed fermented *S. oleraceus Jiangshui*; F3 is traditional starter culture (*Jiangshui Yinzi*) fermented *S. oleraceus Jiangshui*; F4 is natural fermented *S. oleraceus Jiangshui* (CK). All values in the table were mean ± standard deviation (*n* = 3). Different lowercase letters in the same row indicate significant differences (*p* < 0.05, one-way ANOVA followed by Tukey’s test).

### 3.3 Fermentation performance of *Sonchus oleraceus Jiangshui*

#### 3.3.1 The changes of pH, total acid and reducing sugar during the fermentation of *Sonchus oleraceus Jiangshui*

**Figure 6:**
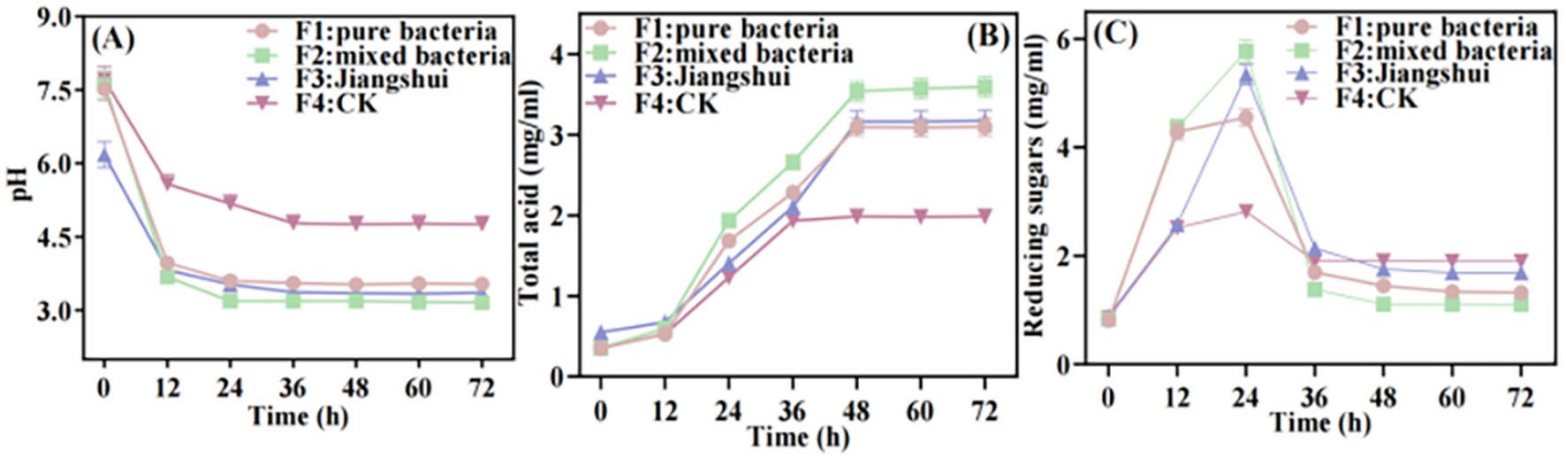
Changes of pH **(A)**, total acid **(B)**, and reducing sugar **(C)** during the fermentation of *S. oleraceus Jiangshui* with different fermentation methods. F1 is LAB 815 fermented *S. oleraceus Jiangshui*; F2 is LAB 815 and SC 8(3) mixed fermented *S. oleraceus Jiangshui*; F3 is traditional starter culture (*Jiangshui Yinzi*) fermented *S. oleraceus Jiangshui*; F4 is natural fermented *S. oleraceus Jiangshui* (CK). All values in the figure were mean ± standard deviation (*n* = 3).

With the increase of fermentation time, the pH value of *S. oleraceus Jiangshui* in F1, F2 and F3 was decreased sharply at 12 h, especially in F2, which decreased sharply from 7.62 to 3.16 at 12 h. In the mature stage of fermentation, the pH values of F1, F2 and F3 were 3.54, 3.16 and 3.36, respectively, which were maintained at 3.16–3.55. The pH value of F4 decreased slowly during fermentation, reaching 4.76 at maturity (Figure 6A). The total acid content in F1, F2, and F3 increased sharply within the first 48 h of fermentation and then stabilized. In particular, F2 showed a marked increase from 0.35 mg/mL to 3.59 mg/mL, which was significantly higher than the levels observed in F1, F3, and F4 (*p* < 0.001). In contrast, the total acid content in F4 increased slowly, rising from 0.35 mg/mL to only 1.98 mg/mL by the end of fermentation (Figure 6B). The reducing sugar content in F1, F2, F3, and F4 increased significantly during the first 24 h of fermentation, followed by a marked decrease between 24 and 36 h, and then tended to stabilize after 36 h. The reducing sugar contents in F2 increased the most during the fermentation process, reached 5.77 mg/mL at 24 h of fermentation, and it decreased to 1.37 mg/mL after being utilized by microorganisms in the later stage of fermentation compared to F1, F3 and F4 from 24 to 36 h. It could be concluded that the reducing sugar content stabilized at 1.1 ± 0.3 mg/mL (Figure 6C). These findings suggest that F2 produced the highest amount of acid during fermentation and exhibited the greatest utilization of reducing sugars.

#### 3.3.2 Metabolite determination of fermented *Sonchus oleraceus Jiangshui*

##### 3.3.2.1 Amino acid analysis

The flavor of *Jiangshui* is primarily influenced by sweet and umami amino acids such as Glu, Asp., Ala, and Gly; therefore, the concentrations of these amino acids were analyzed (Table 5). In this study, seventeen free amino acids were detected in *S. oleraceus Jiangshui*. Our study found that the content of these sweet and umami amino acids increased significantly after inoculated fermentation with probiotics (*p* < 0.001, Table 5). Especially, the content of sweet and umami amino acids was the highest in F2 (250.4 mg/100 mL), which was significantly higher than that in F0 (5.29 mg/100 mL, *p* < 0.001). However, the content of sweet and umami amino acids in F4 group did not increase significantly, but the bitter amino acid in F4 group (92.8 mg/100 mL) was significantly higher than that in other groups (*p* < 0.001). At the end of fermentation, the total amino acid content of F2 (348.1 mg/100 mL) and F3 (325.2 mg/100 mL) was significantly higher than that of F1 (109.2 mg/100 mL), F4 (110.24 mg/100 mL), and F0 (8.146 mg/100 mL) (*p* < 0.001). No significant difference was observed between F2 and F3. It is worth noting that the total amino acid content in F1, F2, F3 and F4 was 13.4-fold, 42.73-fold, 39.9-fold and 13.53-fold of that before fermentation (F0), respectively (Table 5). No Met was produced before and after fermentation of *S. oleraceus.* However, Tyr was found in F1, F2, F3, F4, but not in F0. It could be concluded that the content of sweet and umami amino acids was the highest in F2, followed by F3, F4 and F1, and the amino acid content increased significantly after fermentation.

**Table 5:** The amino acid content (mg/100 mL) of inoculated fermentation, natural fermentation and unfermented *S. oleraceus Jiangshui*.

Amino acid types	Amino acid content(mg/100 mL)					
	F0	F1	F2	F3	F4	Characteristic flavors
Ser	0.41^d^	2.1^c^	53^b^	65.1^a^	3.5^d^	Sweet
Gly	0.28^d^	11.3^bc^	12.2^b^	13.7^a^	2.8^d^	
Ala	ND	ND	ND	52.2^a^	4.6^b^	
His	0.21^d^	9.4^bc^	11.1^b^	15.6^a^	1.5^d^	
Pro	0.91^d^	6.7^c^	56.7^a^	11.2^b^	5.4^d^	
Thr	0.29^d^	2.1^c^	42.7^a^	4.9^b^	3.7^d^	
Total	2.1^e^	31.6^c^	175.7^a^	162.7^b^	21.5^d^	
Asp	0.39^d^	12.7^c^	43.5^b^	56.8^a^	0.59^d^	Fresh
Glu	2.8^d^	21.1^bc^	31.2^a^	22.2^b^	1.3^d^	
Total	3.19^d^	33.8^c^	74.7^ab^	79^a^	1.89^e^	
Cys	0.046^d^	1.3^c^	23.1^b^	31.1^a^	0.096^d^	Bitter
Val	0.36^d^	1.8^bc^	2.3^b^	4.8^a^	0.33^d^	
Met	ND	ND	ND	ND	ND	
His	0.21^e^	9.4^d^	11.1^bc^	12.2^b^	50.15^a^	
Ile	0.29^d^	5.1^a^	1.5^bc^	2.6^b^	0.22^d^	
Leu	0.52^e^	2.4^d^	13.3^b^	5.8^c^	40.41^a^	
Lys	0.21^d^	1.8^c^	12.6^b^	15.1^a^	0.42^d^	
Phe	0.95^d^	8.1^b^	9.4^a^	1.1^c^	0.96^d^	
Arg	0.27^b^	ND	ND	4.3^a^	0.29^b^	
Total	2.856^e^	29.9^d^	73.3^bc^	77^b^	92.8^a^	
Tyr	ND	13.9^b^	24.4^a^	6.5^d^	13.4^bc^	Astringent
Total amino acids	8.146^c^	109.2^b^	348.1^a^	325.2^a^	110.24^b^	―

F0 is unfermented *S. oleraceus Jiangshui*; F1 is LAB 815 fermented *S. oleraceus Jiangshui*; F2 is LAB 815 and SC 8(3) mixed fermented *S. oleraceus Jiangshui*; F3 is traditional starter culture (*Jiangshui Yinzi*) fermented *S. oleraceus Jiangshui*; F4 is natural fermented *S. oleraceus Jiangshui* (CK). All values in the table were mean ± standard deviation (*n* = 3). Different lowercase letters in the same row indicate significant differences (*p* < 0.05, one-way ANOVA followed by Tukey’s test).

##### 3.3.2.2 Analysis of volatile compounds

Volatile compounds was an important indicator of quality and taste of *Jiangshui* products, therefore, the volatile compounds of *S. oleraceus Jiangshui* were identified and quantified in the present study. It was found that there was a total of 45 volatile compounds in the samples of all *S. oleraceus Jiangshui*, including 9 organic acids, 5 terpenoids, 6 alcohols, 4 aldehydes, 4 ketones, 14 esters and 3 else (Table 6). In the present study, 24 new volatile flavor compounds were identified in F2 compared to F0, and the content of alcohols was the highest (29.91%) of the total mass of *Jiangshui*, followed by esters (25.295%) and organic acids (16.36%). Compared with F0, it increased by 29.118, 25.295 and 15.12%, respectively. Esters accounted for only 4.4% of the total volatile compounds in F0, F1, and F4. In contrast, ketones, terpenes, and aldehydes together comprised 10.05% of the total mass of *Jiangshui* The total volatile substances of F1, F2, F3 and F4 increased by 17.398, 82.028, 45.778 and 6.998% compared with F0, respectively. Phenethyl alcohol (+26.75%), damascenone (+0.359%) and damascenone (+ 0.706%) were also detected in F2. Ketones, aldehydes, and terpenes were not detected in the F1 group, while terpenes, aldehydes, and other compounds were not detected in the F4 group (Table 6). The overall results indicated that the content of volatile substances related to the flavor of *S. oleraceus Jiangshui* increased significantly after fermentation, and the content of volatile substances in F2 was significantly higher than that in other methods (*p* < 0.001).

**Table 6:** The content of volatile compounds in inoculated fermentation, natural fermentation *S. oleraceus Jiangshui* and unfermented *S. oleraceus Jiangshui* (%).

Types	Component	CAS	Relative content/%				
			F0	F1	F2	F3	F4
Organic acid	Da	2316-26-9	0.174^a^	ND	ND	ND	ND
	Propionic acid	77-68-9	1.066^a^	ND	ND	ND	ND
	Caprylic acid	124-07-2	ND	1.778^bc^	7.751^a^	1.895^b^	0.651^d^
	Caproic acid	142-62-1	ND	0.501^a^	ND	ND	0.353b
	Decanoic acid	334-48-5	ND	ND	6.076^a^	2.590^b^	ND
	Lauric acid	143-07-7	ND	ND	2.534^a^	0.470^b^	0.249^bc^
	Valeric acid	109-52-4	ND	ND	ND	0.279^a^	ND
	Nonanoic acid	112-05-0	ND	ND	ND	0.983^a^	ND
	Isovaleric acid	503-74-2	ND	ND	ND	ND	0.224^a^
	Total	―	1.24^e^	2.279^c^	16.36^a^	6.217^b^	1.477^d^
Terpenoids	Ut	62951-96-6	ND	ND	0.228^a^	ND	ND
	Geranylacetone	3796-70-1	ND	ND	1.673^a^	0.378^b^	ND
	6,8-Nonadien-2-one	60714-16-1	ND	ND	0.336^a^	ND	ND
	1,1,4,7-Tetramethylindane	1078-04-2	ND	ND	0.088^a^	ND	ND
	Total	―	0	0	2.325^a^	0.378^b^	0
Esters	Ethyl acetate	141-78-6	ND	ND	5.585^a^	ND	ND
	Phenethyl acetate	103-45-7	ND	ND	4.22^ab^	4.465^a^	ND
	Propyl decanolactone	706-14-9	ND	1.782^b^	5.675^a^	ND	0.824^c^
	Ethyl caprylate	106-32-1	ND	ND	0.540^a^	ND	ND
	Ethyl phenylacetate	101-97-3	ND	ND	0.313^a^	ND	ND
	Phenethyl acetate	103-45-7	ND	ND	2.700^a^	ND	1.794^b^
	Benzoic acid ethyl ester	66315-23-9	ND	ND	0.252^a^	ND	ND
	Ethyl decanoate	110-38-3	ND	ND	2.890^a^	ND	ND
	Ethyl laurate	106-33-2	ND	ND	1.364^a^	0.197^b^	ND
	Ethyl palmitate	628-97-7	ND	ND	0.532^b^	0.728^a^	ND
	Eo	544-35-4	ND	ND	0.368^a^	ND	ND
	Ethyl linolenate	1191-41-9	ND	ND	0.856^a^	0.369^b^	ND
	Ethyl 2-phenylcaprylate	5457-70-5	ND	ND	ND	0.105^a^	ND
	Butyl isobutyrate	97-87-0	ND	ND	ND	0.187^a^	ND
	Total	―	0	1.782^d^	25.295^a^	6.051^b^	2.618^c^
Alcohols	Diethylene glycol	111-46-6	0.792^a^	ND	ND	ND	ND
	Phenylethanol	60-12-8	ND	15.37^c^	26.75^a^	18.46^b^	4.136^d^
	Np	6308-98-1	ND	ND	3.159^a^	ND	ND
	Trans-2-Nonen-1-ol	31502-14-4	ND	ND	ND	0.631^a^	ND
	Alpha-terpineol	10482-56-1	ND	ND	ND	3.427^a^	0.113^b^
	3-Cyclohexyl-1-propanol	1124-63-6	ND	ND	ND	0.515^a^	ND
	(−)-4 - Terpineol	20126-76-5	ND	ND	ND	1.227a	ND
	Total	―	0.792^e^	15.37^c^	29.91^a^	24.26^b^	4.249^d^
Aldehydes	3,4-Dimethylbenzaldehyde	5973-71-7	ND	ND	6.664^a^	ND	ND
	Trans-2-Nonenal	18829-56-6	ND	ND	ND	0.198^a^	ND
	Trans-2-Decenal	3913-81-3	ND	ND	ND	1.141^a^	ND
	Peach aldehyde	104-67-6	ND	ND	ND	1.064^a^	ND
	Total	―	0	0	6.664^a^	2.403^b^	0
Tones	Damascenone	23726-91-2	ND	ND	0.359^a^	ND	ND
	Damarone	23726-93-4	ND	ND	0.706^a^	0.177^c^	0.683^ab^
	3-Hydroxy-2-butanone	513-86-0	ND	ND	ND	8.189^a^	ND
	3-Hydroxyacetophenone	121-71-1	ND	ND	ND	0.137^a^	ND
	Total	―	0	0	1.065^b^	8.503^a^	0.683^c^
Else	1,2,3-Trimethyl 1H-indole	1971-46-6	ND	ND	0.049^a^	ND	ND
	1-Ethyl-2-phenylhydrazine	622-82-2	ND	ND	0.307^a^	ND	ND
	Tt	475-03-6	ND	ND	2.082^a^	ND	ND
	Total	―	0	0	2.438^a^	0	0
―	Total Volatile substance	―	2.032^e^	19.43^c^	84.06^a^	47.81^b^	9.03^d^

Da is 3,4-Dimethoxycinnamic acid; Ut is1,5,9-Undecatriene, 2,6,10-trimethyl-, (Z)-; Eo is Ethyl (Z, Z)-9,12-octadecadienoate; Np is N-(2-phenylethyl) phenethylamine; Tt is 1,2,3,4-tetrahydro-1,1,6-trimethylnaphtha. F0 is unfermented *S. oleraceus Jiangshui*; F1 is LAB 815 fermented *S. oleraceus Jiangshui*; F2 is LAB 815 and SC 8(3) mixed fermented *S. oleraceus Jiangshui*; F3 is traditional starter culture (*Jiangshui Yinzi*) fermented *S. oleraceus Jiangshui*; F4 is natural fermented *S. oleraceus Jiangshui* (CK). All values in the table were mean (*n* = 3). Different lowercase letters in the same row indicate significant differences (*p* < 0.05, one-way ANOVA followed by Tukey’s test).

#### 3.3.3 Sensory evaluation

**Figure 7:**
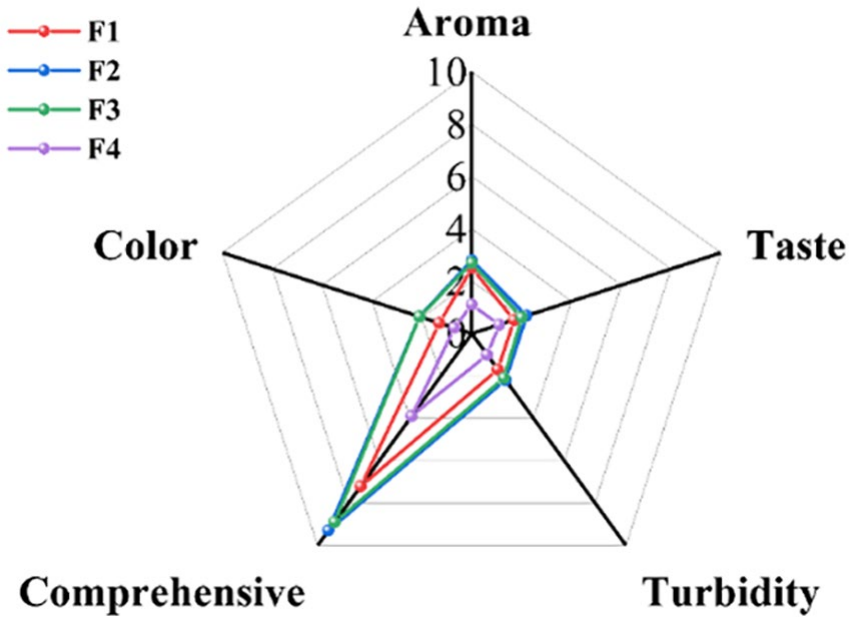
Effect of different fermentation methods on sensory attributes of *S. oleraceus Jiangshui*. F1 is LAB 815 pure bacterial fermented *S. oleraceus Jiangshui*; F2 is LAB 815 and SC 8(3) mixed fermented *S. oleraceus Jiangshui*; F3 is traditional starter culture (*Jiangshui Yinzi*) fermented *S. oleraceus Jiangshui*; F4 is natural fermented *S. oleraceus Jiangshui* (CK): Juice is fresh *S. oleraceus* juice. All values in the figure were mean ± standard deviation (*n* = 3).

The sensory characteristics of four fermented *S. oleraceus Jiangshui*. were shown in Figure 7. The total sensory evaluation scores of F1, F2, F3 and F4 were 7.2, 9.3, 8.9 and 3.9, respectively. Compared with F4, F2 had the highest scores in aroma (2.8 points), taste (2.2 points), Turbidity (2.2 points), and color (2.1 points) (Figure 7). The current results show that the sensory characteristics of *S. oleraceus* can be improved after fermentation with LAB 815 and SC 8 (3).

## 4 Discussion

The *S. oleraceus* is recognized for its rich phytochemical diversity and has been traditionally used in folk medicine to treat various metabolic diseases (Rolnik and Olas, 2021). Laabar et al. (2025) reported that *S. oleraceus* exhibited significant *α*-glucosidase and α-amylase inhibitory activities, along with notable antioxidant properties. Similarly, Du et al. (2024) demonstrated that probiotic fermentation enhances the production of bioactive compounds, thereby improving the antioxidant, hypoglycemic, and hypotensive effects of *Porphyra yezoensis*. Currently, probiotics are recognized as promising therapeutic agents for treating metabolic disorders and restoring imbalanced intestinal ecosystems (Upadrasta and Madempudi, 2016). A study by Chakravarty et al. (2025) showed that LAB NZ9000 effectively reduced blood glucose. Wang G. et al. (2020) obtained similar results, found that *Bifidobacterium adolescentis*, *B. bifidum* and *Lactobacillus rhamnosus* can reduce fasting and postprandial blood glucose levels in type 2 diabetic mice. Several studies also have shown that probiotics (*Lactobacillus casei*) can control blood pressure levels by improving lipid levels, triglyceride levels, bile acid hydrolysis binding, body mass index control and other mechanisms of cross-linking (Lakshmanan et al., 2022; Moiseenko et al., 2023). Therefore, this study aimed to utilize endogenous probiotics from the *Jiangshui* source as fermentation starters to ferment *S. oleraceus*, hypothesizing that the combined approach may exhibit enhanced functional activities relevant to diabetes and hypertension management compared to using probiotics or *S. oleraceus* alone. In this study, LAB and yeast were co-fermented with *S. oleraceus* to produce *S. oleraceus Jiangshui*, and its potential *in vitro* effects on *α*-glucosidase, α-amylase, and ACE inhibitory activities were systematically evaluated.

The beneficial properties of probiotics have always been the focus of attention, The survival of probiotics in the gastrointestinal environment is the premise to exert beneficial characteristics (Liong and Shah, 2005; Sarita et al., 2025). The current study reported that the probiotic characteristics in this study showed that the high survival rates of strains LAB 815 and 8 (3) in simulated gastric fluid (84.15 and 80.21%) and simulated intestinal fluid (78.24 and 76.25%) indicated that the two strains all had strong tolerance to acid and bile salts (Table 3). Our results are consistent with the findings of Wang et al. (2021) who reported that *Lactobacillus fermentum* 21,828 exhibited strong tolerance to pH 2.5 and bile salts. Similarly, Aarti et al. (2017) found that *Lactobacillus brevis* strain LAP2 could survive under low pH conditions and in the presence of 0.3% (w/v) bile salts. In addition, Jin et al. (2024) demonstrated that *Pediococcus* strains JS 91 and JS 96, isolated from *Jiangshui*, showed high resistance to both acidic environments and bile salts. The current study reported that the hydrophobicity of LAB 815 and SC 8 (3) were 34.03 and 36.85% respectively, and the auto-aggregation of LAB 815 and SC 8 (3) were 27.24 and 20.88%, respectively (Table 3), which indicated that LAB 815 and SC 8 (3) could adhere to the surface of the host mucosa and thereby exerting its probiotic effect. Similar findings were also stated that hydrophobicity and auto-aggregation are prerequisites for LAB to adhere to intestinal epithelial cells and play a probiotic role (Beldarrain-Iznaga et al., 2021). However, excessive self-polymerization may also lead to uneven distribution of LAB in the intestine and affect its probiotic effect. *Lactobacillus plantarum* L7 isolated from traditional rice fermented beverages reported by Giri et al. (2018) showed high hydrophobicity and strong auto-aggregation ability. Hemolytic activity is regarded as a key safety criterion for the selection of probiotic strains (FAO/WHO, 2006). In this study, both LAB 815 and SC 8(3) showed no hemolytic activity (*γ*-hemolysis) on blood agar plates, indicating that they pose no risk of damaging red blood cells (Figure 1). Our results were in agreement with those reported by Wang et al. (2018) regarding *Lactobacillus plantarum* and *Weissella cibaria* strains isolated from spontaneously fermented non-dairy foods in China. These results demonstrate that LAB 815 and SC 8(3) exhibit good gastrointestinal tolerance, adhesion ability, and safety.

Several studies have shown that *α*-amylase and α-glucosidase were involved in carbohydrate metabolism and were related to postprandial glucose levels (Teugwa et al., 2013; Laabar et al., 2025). It was reported that *α*-glucosidase, α-amylase inhibition rate and GDRI are the key indicators of *in vitro* hypoglycemic ability (Huligere et al., 2023; Chen et al., 2021). Similarly, Qi et al. (2024) reported that after probiotic fermentation of wolfberry juice, the hypoglycemic ability of wolfberry juice was improved, and the inhibition rates of α-glucosidase and α-amylase reached 52.30 and 51.79%, respectively. Wang W. L. et al. (2020) found that the hypoglycemic activity of Aksu apple juice fermented by *Lactobacillus fermentum* was significantly increased. Pan et al. (2025) reported that the GDRI of loquat juice fermented by *L. brevis* was the highest after dialysis for 30 min and 60 min, which was 16.49 and 9.76%, respectively. In this study, both α-glucosidase and α-amylase activities were inhibited by all four types of *S. oleraceus Jiangshui* as well as by *S. oleraceus* juice. Notably, the inhibition rates of both enzymes were significantly enhanced following probiotic fermentation. Compared with *S. oleraceus* juice, the inhibition rates of α-glucosidase and α-amylase in F2 were significantly increased to 75.14 and 68.14%, respectively, reaching 91.27 and 81.77% of the acarbose inhibition effect, respectively (*p* < 0.001, Figures 2A,B). Also, the GDRI of F2 exhibited the highest GDRI (55.67%), which was 2.36-fold higher than that of *S. oleraceus* juice (Figure 2C). These results showed that co-fermentation of *S. oleraceus Jiangshui* with LAB 815 and SC 8(3) enhanced its α-glucosidase and α-amylase inhibitory activities *in vitro*. Although the inhibitory effects were lower than those of acarbose, the fermented product still exhibits potential as a functional food for postprandial blood glucose regulation.

The determination of ACE activity is a key step in evaluating the antihypertensive potential of the tested substances, and the inhibition rate of ACE activity is significantly correlated with the hypotensive properties (Bao and Chi, 2016). The current study showed that the *S. oleraceus Jiangshui* co-fermented with LAB 815 and yeast 8 (3) had the highest ACE inhibition rate (86.86%), followed by F1 and F3, reached 69.8 and 71%, respectively (Figure 3). This study is in consistent with a recent study which indicated that the incorporation of probiotics can significantly increase the inhibition rate of ACE activity and improved the hypotensive properties (Moiseenko et al., 2023). The ACE inhibition rate significantly increased after microbial fermentation compared with the fresh *S. oleraceus* juice, indicating that the ACE inhibitory activity of *S. oleraceus Jiangshui* was enhanced by probiotic fermentation (*p* < 0.001). The α-glucosidase, α-amylase and ACE activity inhibition rate decreased *in vitro* simulated gastrointestinal fluid experiments. In particular, the α-glucosidase inhibition rate decreased from 75.14 to 50.52% post-digestion, representing a 32.8% reduction in activity in group F2; the α-amylase inhibition rate decreased from 68.14 to 56.37% (a 17.3% reduction); and the ACE inhibition rate decreased from 86.86 to 68.72% (a 20.9% reduction). This decline is likely due to the degradation of bioactive peptides by gastrointestinal proteases, which cleave peptide bonds at specific residues and reduce their functional activity (Chen et al., 2025). However, the inhibition rates of α-glucosidase, α-amylase, and ACE inhibition rates in F1, F2, and F3 remained higher after fermentation compared to their pre-fermentation levels. Among them, F2 exhibited the highest inhibition rates for all three enzymes (Figure 4). These results indicated that LAB 815 and SC 8(3) cooperate with each other, which greatly reduces the antagonistic effect in the high acid environment of the gastrointestinal tract.

Previous studies reported that LAB fermentation can increase the *in vitro* antioxidant and antibacterial activities of fermented foods (Rocchetti et al., 2019; Chen et al., 2020; Hashemi et al., 2021). Zhang et al. (2024) shown that organic acids (lactic acid, acetic acid, etc.) and flavonoids in *Jiangshui* effectively inhibit the growth of some spoilage bacteria. So, the influences of LAB 815 and SC 8(3) co-fermentation on antioxidant activities and antibacterial activity of *S. oleraceus Jiangshui* were further investigated. The present study showed that all the four tested *S. oleraceus Jiangshui* had free radical scavenging ability and inhibit pathogenic bacteria ability. Moreover, F2 had the greatest scavenging ability of DPPH free radical, hydroxyl radical and superoxide anion radical compared to other fermentation methods (Figure 5), while F2 also had the strongest inhibitory effect on *Staphylococcus aureus*, *Escherichia coli*, and *Klebsiella pneumoniae* (Table 4). These results indicated that LAB 815 and SC 8(3) co-fermentation could markedly influenced the antioxidant activity and antibacterial activity of *S. oleraceus Jiangshui*. The antibacterial activity of *S. oleraceus Jiangshui* may be closely related to the acidic substances and bacteriocins produced by lactic acid bacteria (Du et al., 2014; Lu et al., 2020). It is speculated that the enhancement of bacteriostatic effect in the process of *Jiangshui* fermentation is related to the proliferation of LAB and the increase of total organic acid in the early and middle stages of fermentation.

The changes of pH value and total acid can reflect the changes of free H^+^ and organic acids formed during the fermentation process, as well as the consumption of substrates for reducing sugar reactions (Chen et al., 2018). Wang et al. (2021) found that, following the addition of lactic acid bacteria to pear juice, pH and total acid content were inversely proportional. Additionally, the reducing sugar content significantly decreased from 64.76 g/L to 47.26–57.53 g/L within 12 h. The present study showed that the pH values of F1, F2, F3, and F4 all exhibited a rapid decline within the first 12 h of fermentation, followed by stabilization. Notably, in F2, the pH dropped sharply from 7.62 to 3.16 within 12 h (Figure 6A), and then decreased slightly until the end of fermentation process, and the total acid of F2 accumulated to 3.59 mg/mL at 48 h (Figure 6B). It is possible that the growth of LAB 815 was inhibited in the later stage of fermentation, so the produced lactic acid no longer increased (Deutscher, 2008). These results are similar to those reported by Jin et al. (2024) regarding the changes in pH during the fermentation of pear juice. The reducing sugar contents significantly increased from 0 to 24 h in *S. oleraceus Jiangshui* fermentation (*p* < 0.001), which was due to the mass reproduction of LAB and the production of protease, lipase and amylase, which increased the reducing sugar content (Zhou et al., 2022). The reducing sugar content in F2 decreased significantly from 5.77 mg/mL to 1.37 mg/mL between 24 and 36 h of *S. oleraceus Jiangshui* fermentation (*p* < 0.001, Figure 6C), likely due to the consumption of reducing sugars by the growth and metabolic activity of LAB strains (Chen et al., 2019). Subsequently, during the final 48 h of *S. oleraceus Jiangshui* fermentation, a slight increase in reducing sugar content was observed, which may be attributed to the production of exopolysaccharides by the probiotics (Ricci et al., 2018).

Amino acids and Volatile flavor compounds were the main contributors to the delicate flavor of fermented vegetables (Wu et al., 2015). In the present study, the levels of umami and sweet amino acids in F1, F2, and F3 were significantly higher than those in the unfermented sample (F0) (*p* < 0.001), with F2 exhibiting the highest overall amino acid content (Table 5). These results are consistent with previous studies which have shown that mixed probiotic fermentation can significantly increase the content of amino acids and volatile compounds in food, thereby enhancing its flavor (Ruan et al., 2022; He et al., 2025). The increase in amino acid content may be due to the fact that the acidity destroys the structure of the vegetables, releasing more proteins and amino acids, as well as the microbial growth utilizes the nutrients in the vegetables for metabolism, further increasing the amino acid content (Wang X. W. et al., 2020). The substances transformed from various microbial metabolites produced during fermentation were the main sources of volatile components in *Jiangshui*. Li et al. (2017) identified a total of 182 metabolites in celery *Jiangshui*, primarily comprising organic acids and amino acids. In the present study, a total of 45 volatile compounds were detected in all *S. oleraceus Jiangshui* samples (Table 6), the content of volatile substances increased significantly after fermentation. Furthermore, totally 24 new volatile flavor compounds were identified in F2 compared to F0, with alcohols (+29.118%), esters (+25.295%), and organic acids (+15.12%) being the predominant categories. Additionally, the increased levels of terpenes, aldehydes and ketones in F2 further enhanced the flavor profile (Table 6). Ester compounds are the main source of fruity and floral aromas of berries. The content of esters in F0, F1 and F4 is only 4.4%, which is related to the lack of LAB 815 or SC 8 (3) (Table 6). Organic acids give the *Jiangshui* a sour taste, while the increased content of phenethyl alcohol (+26.75%), damascenone (+0.359%) and damarone (+0.706%) in F2 can give the *Jiangshui* a unique aromatic odor (Table 6). In the study of sensory characteristics, the aroma, taste, color, turbidity and total score of F1, F2 and F3 were higher compared with F4 (CK), and the comprehensive score of F2 was the highest (9.3 points), which seemed to be consistent with the performance of aroma (Figure 7). Therefore, the better smell in the process of fermented *S. oleraceus Jiangshui* may be due to the more flavor substances released in the process of LAB and yeast fermentation, which improved the flavor of *S. oleraceus* itself by producing aroma components such as alcohols, esters and organic acids (Wang et al., 2021). These results indicated that *S. oleraceus Jiangshui* has a better flavor by the co-fermentation of LAB 815 and SC 8 (3). Our study demonstrated that co-fermentation of *S. oleraceus* with LAB 815 and SC 8(3) enhances the fermentation performance and functional properties of *S. oleraceus Jiangshui*, particularly through inhibition of *α*-glucosidase, α-amylase, and ACE activities. These findings highlight its potential as a functional food ingredient for enzyme inhibitory activities related to hyperglycemia and hypertension. However, certain limitations remain as the observed *in vitro* enzyme inhibitory effects have not yet been validated *in vivo* or clinically, necessitating further research to confirm these results.

## 5 Conclusion

In this study, both *Lactobacillus* strain LAB 815 isolated from Gansu *Jiangshui*, and the aroma-producing yeast SC 8(3) demonstrated strong acid and bile salt tolerance, high adhesion potential, and the ability to withstand adverse gastrointestinal conditions, enabling them to function effectively within the digestive tract. Additionally, neither strain exhibited hemolytic activity, confirming their safety as potential probiotics. The combined fermentation of *S. oleraceus* with LAB 815 and SC 8(3) resulted in the formation of *S. oleraceus Jiangshui*, which exhibited significant inhibitory effects on α-amylase, α-glucosidase, and ACE activities. It also showed the highest glucose diffusion retardation index (GDRI), antioxidant activity, and antibacterial efficacy among the tested samples. Simulated gastrointestinal digestion further confirmed that the enzyme inhibitory activities related to glycemic and blood pressure regulation were best preserved in the co-fermented product, suggesting synergistic interactions between LAB 815 and SC 8(3) that enhance stability under gastrointestinal conditions. Moreover, co-fermentation significantly enhanced the amino acid and volatile compound profiles of *S. oleraceus Jiangshui*, resulting in an improved flavor profile. Overall, these findings demonstrate that the combined fermentation with LAB 815 and SC 8(3) not only enhances the functional bioactivities but also improves the sensory qualities of *S. oleraceus Jiangshui*, underscoring its potential as a promising strategy for developing functional fermented foods with enhanced health benefits and consumer appeal.

## Data Availability

The original contributions presented in the study are included in the article/supplementary material, further inquiries can be directed to the corresponding authors.

## References

[ref1] Aarti C. Khusro A. Varghese R. Arasu M. V. Agastian P. Al-Dhabi N. A. . (2017). *In vitro* studies on probiotic and antioxidant properties of *Lactobacillus brevis* strain LAP2 isolated from Hentak, a fermented fish product of north-East India. LWT 86, 438–446. doi: 10.1016/j.lwt.2017.07.055

[ref2] Alothman E. A. Awaad A. S. Safhi A. A. Almoqren S. S. El-Meligy R. M. Zain Y. M. . (2018). Evaluation of anti-ulcer and ulcerative colitis of *Sonchus Oleraceus* L. Saudi Pharm. J. 26, 956–959. doi: 10.1016/j.jsps.2018.05.004, 30416352 PMC6218846

[ref3] Bao Z. J. Chi Y. J. (2016). *In vitro* and *in vivo* assessment of angiotensin-converting enzyme (ACE) inhibitory activity of fermented soybean Milk by *Lactobacillus casei* strains. Curr. Microbiol. 73, 214–219. doi: 10.1007/s00284-016-1051-7, 27139252

[ref4] Beldarrain-Iznaga T. Villalobos-Carvajal R. Sevillano-Armesto E. Leiva-Vega J. (2021). Functional properties of *Lactobacillus casei* C24 improved by microencapsulation using multilayer double emulsion. Food Res. Int. 141:110136. doi: 10.1016/j.foodres.2021.110136, 33642003

[ref5] Caffrey E. B. Sonnenburg J. L. Devkota S. (2024). Our extended microbiome: the human-relevant metabolites and biology of fermented foods. Cell Metab. 36, 684–701. doi: 10.1016/j.cmet.2024.03.007, 38569469 PMC12220828

[ref6] Chakravarty K. Gaur S. Kumar R. Jha N. K. Gupta P. K. (2025). Exploring the multifaceted therapeutic potential of probiotics: a review of current insights and applications. Probiotics Antimicrobial Proteins 17, 341–363. doi: 10.1007/s12602-024-10328-x, 39069588

[ref7] Chen R. H. Chen W. X. Chen H. M. Zhang G. F. Chen W. J. (2018). Comparative evaluation of the antioxidant capacities, organic acids, and volatiles of papaya juices fermented by *Lactobacillus acidophilus* and *Lactobacillus plantarum*. J. Food Qual. 2018, 1–12. doi: 10.1155/2018/9490435

[ref8] Chen J. W. Meng Q. Jiang B. Chen J. J. Zhang T. Zhou L. C. (2021). Structure characterization and *in vitro* hypoglycemic effect of partially degraded alginate. Food Chem. 356:129728. doi: 10.1016/j.foodchem.2021.129728 33836362

[ref9] Chen T. Piao M. Rahman S. M. E. Zhang L. Deng Y. (2020). Influence of fermentation on antioxidant and hypolipidemic properties of maifanite mineral water-cultured common buckwheat sprouts. Food Chem. 321:126741. doi: 10.1016/j.foodchem.2020.126741, 32276146

[ref10] Chen P. Wu Z. J. Zhao Y. Wei Y. Xu R. X. Yan L. . (2015). Cultivation-independent comprehensive investigations on bacterial communities in Serofluid dish, a traditional Chinese fermented food. Genomics Data 7, 127–128. doi: 10.1016/j.gdata.2015.12.019, 26981386 PMC4778640

[ref11] Chen H. Xiao G. Xu Y. Yu Y. Wu J. Zou B. (2019). High hydrostatic pressure and co-fermentation by Lactobacillus rhamnosus and *Gluconacetobacter xylinus* improve flavor of yacon-litchi-longan juice. Foods 8:308. doi: 10.3390/foods8080308, 31374950 PMC6722649

[ref12] Chen C. L. Yu W. C. Kou X. H. Niu Y. J. Ji J. X. Shao Y. . (2025). Recent advances in the effect of simulated gastrointestinal digestion and encapsulation on peptide bioactivity and stability. Food Funct. 16, 1634–1655. doi: 10.1039/d4fo04447a, 39943857

[ref13] Cheng Z. X. Yang J. H. Yan R. Y. Wang B. W. Bai Y. Miao Z. J. . (2025). Interactive mechanism-guided microbial interaction dynamics in food fermentations: lactic acid bacteria and yeasts as a case example. Food Biosci. 68:106453. doi: 10.1016/j.fbio.2025.106453

[ref14] Couto V. M. Vilela F. C. Dias D. F. Santos M. H. d. Soncini R. Nascimento C. G. O. . (2011). Antinociceptive effect of extract of *Emilia sonchifolia* in mice. J. Ethnopharmacol. 134, 348–353. doi: 10.1016/j.jep.2010.12.028, 21185930

[ref15] Cruz-Huerta E. Garcia-Nebot M. J. Miralles B. Recio I. Amigo L. (2014). Caseinophosphopeptides released after tryptic hydrolysis versus simulated gastrointestinal digestion of a casein-derived by-product. Food Chem. 168, 648–655. doi: 10.1016/j.foodchem.2014.07.090, 25172759

[ref16] Deutscher J. (2008). The mechanisms of carbon catabolite repression in bacteria. Curr. Opin. Microbiol. 11, 87–93. doi: 10.1016/j.mib.2008.02.007, 18359269

[ref17] Dou Z. M. Chen C. Fu X. (2019). The effect of ultrasound irradiation on the physicochemical properties and α-glucosidase inhibitory effect of blackberry fruit polysaccharide. Food Hydrocoll. 96, 560–576. doi: 10.1016/j.foodhyd.2019.06.002

[ref18] Du X. Li L. Liu D. M. (2014). Effect of carbon source on antioxidant and bacteriostatic activities of soybean whey fermented by probiotic bacterium. Modern Food Sci. Technol. 30, 129–133. doi: 10.13982/j.mfst.1673-9078.2014.02.034

[ref19] Du Y. L. Wang L. Xu J. C. Gao X. Fu X. T. (2024). Active components and antioxidant, hypoglycaemic and antihypertensive activities of fermented *Pyropia yezoensis*. Sci. Technol. Food Ind. 45, 72–80. doi: 10.13386/j.issn1002-0306.2023120247

[ref20] FAO/WHO (2006). Probiotics in Food: Health and Nutritional Properties and Guidelines for Evaluation. FAO Food Nutrition Paper 85. Rome: World Health Organization and Food and Agriculture Organization of the United Nations.

[ref21] Galena A. E. Chai J. Zhao J. Bednarzyk M. Perez D. Ochrietor J. D. . (2024). The effects of fermented vegetable consumption on the composition of the intestinal microbiota and levels of inflammatory markers in women: a pilot and feasibility study. PLoS One 19:e0306219. doi: 10.1371/journal.pone.0275275, 38913682 PMC11195968

[ref22] Giri S. S. Sen S. S. Saha S. Sukumaran V. Chang P. S. (2018). Use of a potential probiotic, *Lactobacillus plantarum* L7, for the preparation of a rice-based fermented beverage. Front. Microbiol. 9:473. doi: 10.3389/fmicb.2018.00473, 29593702 PMC5861207

[ref23] Guo W. Chen M. Cui S. Tang X. Zhang Q. Zhao J. . (2023). Effects of *Lacticaseibacillus casei* fermentation on the bioactive compounds, volatile and non-volatile compounds, and physiological properties of barley beverage. Food Biosci. 53:102695. doi: 10.1016/j.fbio.2023.102695

[ref24] Hashemi S. M. B. Jafarpour D. Jouki M. (2021). Improving bioactive properties of peach juice using Lactobacillus strains fermentation: antagonistic and anti-adhesion effects, anti-inflammatory and antioxidant properties, and Maillard reaction inhibition. Food Chem. 365:130501. doi: 10.1016/j.foodchem.2021.130501, 34247050

[ref25] He Z. J. Cao L. J. Zhao L. F. Zhou Y. Gong J. S. Zhu C. . (2025). Effects of the mixed fermentation of apples and yeast on the sensory evaluation, physicochemical composition and flavor of coffee beans. Food Biosci. 68:106337. doi: 10.1016/j.fbio.2025.106337

[ref26] Hu Y. Zhao Y. Jia X. Liu D. Huang X. H. Wang C. . (2023). Lactic acid bacteria with a strong antioxidant function isolated from "jiangshui," pickles, and feces. Front. Microbiol. 14:1163662. doi: 10.3389/fmicb.2023.1163662, 37293224 PMC10246737

[ref27] Huligere S. S. Chandana K. V. B. Desai S. M. Wong L. S. Firdose N. Ramu R. (2023). Investigating the antidiabetic efficacy of dairy-derived *Lacticaseibacillus paracasei* probiotic strains: modulating Α-amylase and Α-glucosidase enzyme functions. Front. Microbiol. 14:1288487. doi: 10.3389/fmicb.2023.1288487, 38111646 PMC10725979

[ref28] Jimoh F. O. Adedapo A. A. Afolayan A. J. (2011). Comparison of the nutritive value, antioxidant and antibacterial activities of *Sonchus asper* and *Sonchus oleraceus*. Rec. Nat. Prod. 5, 29–42. doi: 10.1080/14786419.2010.534093

[ref29] Jin D. X. Jin Y. X. Zhang W. Cao W. Liu R. Wu M. G. . (2024). Exploring Jiangshui-originated probiotic lactic acid bacteria as starter cultures: functional properties and fermentation performances in pear juice. Food Biosci. 61:104982. doi: 10.1016/j.fbio.2024.104982

[ref30] Khan A. Wang W. D. Ji J. Ling Z. M. Liu P. Xiao S. . (2023). Fermented lily bulbs by “jiangshui” probiotics improves lung health in mice. Food Chem. 440:138270. doi: 10.1016/j.foodchem.2023.138270, 38150908

[ref31] Koyuncu G. Cabaroglu T. (2024). Impact of lactic acid bacteria starter inoculation on the physico-chemical, microbiological, volatiles and sensory properties of natural black Gemlik (*Olea europaea* L., cv.) table olives. Food Biosci. 60:104310. doi: 10.1016/j.fbio.2024.104310

[ref32] Laabar A. Kabach I. El Asri S. Kchikich A. Drioua S. El Hamri A. . (2025). Investigation of antioxidant, antidiabetic, and antiglycation properties of *Sonchus oleraceus* and *Lobularia maritima* (L.) Desv. Extracts from Taza, Morocco. Food Chem. Adv. 6:100912. doi: 10.1016/j.focha.2025.100912

[ref33] Lakshmanan A. P. Murugesan S. Al Khodor S. Terranegra A. (2022). The potential impact of a probiotic: *akkermansia muciniphila* in the regulation of blood pressure—the current facts and evidence. J. Transl. Med. 20, 1–15. doi: 10.1186/s12967-022-03631-0, 36153618 PMC9509630

[ref34] Li Q. Q. Kang J. M. Ma Z. Li X. P. Liu L. Hu X. Z. (2017). Microbial succession and metabolite changes during traditional serofluid dish fermentation. LWT-Food Sci. Technol. 84, 771–779. doi: 10.1016/j.lwt.2017.06.051

[ref35] Liang T. T. Xie X. Q. Wu L. Li L. Y. Li H. X. Xi Y. . (2022). Microbial communities and physiochemical properties of four distinctive traditionally fermented vegetables from North China and their influence on quality and safety. Foods 11:21. doi: 10.3390/foods11010021, 35010147 PMC8750469

[ref36] Liong M. T. Shah N. P. (2005). Acid and bile tolerance and cholesterol removal ability of lactobacilli strains. J. Dairy Sci. 88, 55–66. doi: 10.3168/jds.s0022-0302(05)72662-x 15591367

[ref37] Liu Y. W. Li H. B. Wang X. F. Zhang G. X. Wang Y. Di D. L. (2011). Evaluation of the free radical scavenging activity of cynomorium songaricum rupr. By a novel DPPH-HPLC method. J. Food Sci. 76, C1245–C1249. doi: 10.1111/j.1750-3841.2011.02392.x, 22416684

[ref38] Lu Z. Dai Y. Q. Rasheed H. A. Wu H. Xia X. D. Dong M. S. (2020). Antimicrobial activity of soy whey fermented by *Lactobacillus plantarum* D1501 and purification and identification of bacteriocin from it. Food Sci. 41, 117–124. doi: 10.7506/spkx1002-6630-20190929-362

[ref39] Moiseenko K. V. Glazunova O. A. Savinova O. S. Shabaev A. V. Fedorova T. V. (2023). Changes in composition of some bioactive molecules upon inclusion of *Lacticaseibacillus paracasei* probiotic strains into a standard yogurt starter culture. Foods 12:4238. doi: 10.3390/foods12234238, 38231606 PMC10705950

[ref40] Nami Y. Bakhshayesh R. V. Jalaly H. M. Lotfi H. Eslami S. Hejazi M. A. (2019). Probiotic properties of *Enterococcus* isolated from artisanal dairy products. Front. Microbiol. 10:300. doi: 10.3389/fmicb.2019.00300, 30863379 PMC6400110

[ref41] Pan S. G. Wang Q. Liu Z. J. Zhang J. Wei S. F. Shuai G. P. . (2025). Effect of lactic acid bacteria fermentation on hypoglycemic and hypolipidemic activity of loquat juice *in vitro*. Sci. Technol. Food Ind. 46, 161–168. doi: 10.13386/j.issn1002-0306.2024050345

[ref42] Park J. Heo S. Lee G. Kim T. Oh S.-E. Kwak M.-S. . (2024). The addition of Jogi, Micropogonias undulates, affects amino acid content in kimchi fermentation. PLoS One 19:e0300249. doi: 10.1371/journal.pone.0300249, 38573994 PMC10994411

[ref43] Pieniz S. Andreazza R. Anghinoni T. Camargo F. Brandelli A. (2014). Probiotic potential, antimicrobial and antioxidant activities of *Enterococcus durans* strain LAB18s. Food Control 37, 251–256. doi: 10.1016/j.foodcont.2013.09.055

[ref44] Qi Y. Wei X. B. Ma C. Liu H. Y. Fang H. T. (2024). Study on *Lycium barbarum* juice fermented by probiotics and its hypoglycemic and antioxidant activities *in vitro*. Food Ferment. Ind. 15, 1612–1626. doi: 10.13995/j.cnki.11-1802/ts.039630

[ref45] Ricci A. Cirlini M. Levante A. Dall’Asta C. Galaverna G. Lazzi C. (2018). Volatile profile of elderberry juice: effect of lactic acid fermentation using *L. plantarum*, *L. rhamnosus* and *L. casei* strains. Food Res. Int. 105, 412–422. doi: 10.1016/j.foodres.2017.11.042 29433231

[ref46] Rocchetti G. Miragoli F. Zacconi C. Lucini L. Rebecchi A. (2019). Impact of cooking and fermentation by lactic acid bacteria on phenolic profile of quinoa and buckwheat seeds. Food Res. Int. 119, 886–894. doi: 10.1016/j.foodres.2018.10.073, 30884729

[ref47] Rolnik A. Olas B. (2021). The plants of the Asteraceae Family as agents in the protection of human health. Int. J. Mol. Sci. 22:3009. doi: 10.3390/ijms22063009, 33809449 PMC7999649

[ref48] Ruan W. Liu J. Li P. Zhao W. Zhang A. Liu S. . (2022). Dynamics of microbial communities, flavor, and physicochemical properties during Ziziphus jujube vinegar fermentation: correlation between microorganisms and metabolites. Foods 11:3334. doi: 10.3390/foods11213334, 36359947 PMC9655239

[ref49] Sadishkumar V. Jeevaratnam K. (2017). *In vitro* probiotic evaluation of potential antioxidant lactic acid bacteria isolated from Idli batter fermented with *Piper betle* leaves. Int. J. Food Sci. Technol. 52, 329–340. doi: 10.1111/ijfs.13284

[ref50] Sarita B. Samadhan D. Hassan M. Z. Kovaleva E. G. (2025). A comprehensive review of probiotics and human health-current prospective and applications. Front. Microbiol. 15:1487641. doi: 10.3389/fmicb.2024.1487641, 39834364 PMC11743475

[ref51] Sevindik O. Guclu G. Agirman B. Selli S. Kadiroglu P. Bordiga M. . (2022). Impacts of selected lactic acid bacteria strains on the aroma and bioactive compositions of fermented gilaburu (*Viburnum opulus*) juices. Food Chem. 378:132079. doi: 10.1016/j.foodchem.2022.132079, 35042105

[ref52] Tan X. Q. Cui F. C. Wang D. F. Lv X. R. Li X. P. Li J. R. (2024). Fermented vegetables: health benefits, defects, and current technological solutions. Foods 13:38. doi: 10.3390/foods13010038, 38201066 PMC10777956

[ref53] Teugwa C. M. Mejiato P. C. Zofou D. Tchinda B. T. Boyom F. F. (2013). Antioxidant and antidiabetic profiles of two African medicinal plants: *Picralima nitida* (apocynaceae) and *Sonchus oleraceus* (asteraceae). BMC Complement. Altern. Med. 13:175. doi: 10.1186/1472-6882-13-175, 23855679 PMC3718716

[ref54] Trivedi D. Jena P. K. Patel J. K. Seshadri S. (2013). Partial purification and characterization of a bacteriocin DT24 produced by probiotic vaginal *Lactobacillus brevis* DT24 and determination of its anti-uropathogenic *Escherichia coli* potential. Probiotics Antimicrob. Proteins 5, 142–151. doi: 10.1007/s12602-013-9132-4 26782739

[ref55] Upadrasta A. Madempudi R. S. (2016). Probiotics and blood pressure: current insights. Integr. Blood Press. Control 9, 33–42. doi: 10.2147/ibpc.s73246 26955291 PMC4772943

[ref56] Vilela F. C. Padilha M. d. M. Santos-e-Silva L. d. Alves-da-Silva G. Giusti-Paiva A. (2009). Evaluation of the antinociceptive activity of extracts of *Sonchus oleraceus* L. in mice. J. Ethnopharmacol. 124, 306–310. doi: 10.1016/j.jep.2009.04.037, 19397974

[ref57] Wang X. W. Han M. Z. Zhang M. N. Wang Y. Ren Y. P. Yue T. L. . (2020). *In vitro* evaluation of the hypoglycemic properties of lactic acid bacteria and its fermentation adaptability in apple juice. LWT-Food Sci. Technol. 136:110363. doi: 10.1016/j.lwt.2020.110363

[ref58] Wang G. Si Q. Yang S. Jiao T. Zhu H. Y. Tian P. J. . (2020). Lactic acid bacteria reduce diabetes symptoms in mice by alleviating gut microbiota dysbiosis and inflammation in different manners. Food Funct. 11, 5898–5914. doi: 10.1039/c9fo02761k, 32572400

[ref59] Wang J. Wang J. Yang K. Liu M. M. Zhang J. Wei X. Y. . (2018). Screening for potential probiotic from spontaneously fermented non-dairy foods based on *in vitro* probiotic and safety properties. Ann. Microbiol. 68, 803–813. doi: 10.1007/s13213-018-1386-3

[ref60] Wang L. Zhang H. Lei H. (2021). Phenolics profile, antioxidant activity and flavor volatiles of pear juice: influence of lactic acid fermentation using three Lactobacillus strains in monoculture and binary mixture. Foods 11, 11–11. doi: 10.3390/foods11010011, 35010138 PMC8750113

[ref61] Wang W. L. Zhou X. R. Liu Y. (2020). Characterization and evaluation of umami taste: a review. Trends Anal. Chem. 127:115876. doi: 10.1016/j.trac.2020.115876

[ref62] Won G. Y. Choi S. I. Park N. Y. Kim J. E. Kang C. H. Kim G. H. (2021). *In vitro* antidiabetic, antioxidant activity, and probiotic activities of *Lactiplantibacillus plantarum* and *Lacticaseibacillus paracasei* strains. Curr. Microbiol. 78, 3181–3191. doi: 10.1007/s00284-021-02588-5, 34213618 PMC8289794

[ref63] Wu J. J. Ma Y. K. Zhang F. F. Chen F. S. (2012). Biodiversity of yeasts, lactic acid bacteria and acetic acid bacteria in the fermentation of "Shanxi aged vinegar", a traditional Chinese vinegar. Food Microbiol. 30, 289–297. doi: 10.1016/j.fm.2011.08.010, 22265314

[ref64] Wu Y. Ye Z. Feng P. Y. Li R. Chen X. Tian X. Z. . (2021). Limosilactobacillus Fermentum JL-3 isolated from “jiangshui” ameliorates hyperuricemia by degrading uric acid. Gut Microbes 13, 1–18. doi: 10.1080/19490976.2021.1897211, 33764849 PMC8007157

[ref65] Wu R. N. Yu M. L. Liu X. Y. Meng L. S. Wang Q. Q. Xue Y. T. . (2015). Changes in flavour and microbial diversity during natural fermentation of suan-cai, a traditional food made in Northeast China. Int. J. Food Microbiol. 211, 23–31. doi: 10.1016/j.ijfoodmicro.2015.06.028, 26159472

[ref66] Xia D. Z. Yu X. F. Zhu Z. Y. Zou Z. D. (2011). Antioxidant and antibacterial activity of six edible wild plants (sonchus Spp.) in China. Nat. Prod. Res. 25, 1893–1901. doi: 10.1080/14786419.2010.534093, 21793765

[ref67] Yongsawas R. Inta A. Kampuansai J. Pandith H. Suwannarach N. Lamyong S. . (2022). Bacterial communities in Lanna Phak-Gard-Dong (pickled mustard green) from three different ethnolinguistic groups in northern Thailand. Biology 11:150. doi: 10.3390/biology11010150, 35053147 PMC8772952

[ref68] Yu H.-S. Jang H. J. Lee N.-K. Paik H.-D. (2019). Evaluation of the probiotic characteristics and prophylactic potential of *Weissella cibaria* strains isolated from kimchi. LWT 112:108229. doi: 10.1016/j.lwt.2019.05.127

[ref69] Zhang Z. C. Liang X. L. Zhang Y. S. Xuan X. N. Ding B. Liu H. N. (2024). The changes of organic acids during the fermentation of Jiangshui and its antibacterial properties and antioxidant capacity *in vitro*. J. Chinese Institute Food Sci. Technol. 4, 159–169. doi: 10.16429/j.1009-7848.2024.04.016

[ref70] Zhang H. Wang Q. Liu H. T. Kong B. H. Chen Q. (2020). *In vitro* growth performance, antioxidant activity and cell surface physiological characteristics of *Pediococcus pentosaceus* R1 and *Lactobacillus fermentum* R6 stressed at different NaCl concentrations. Food Funct. 11, 6376–6386. doi: 10.1039/c9fo02309g, 32613220

[ref71] Zhang X. B. Zhu G. P. Zhu Z. X. Nan W. Z. Wu Z. P. (2017). Determination of the polysaccharide content of sweet potato leaves by using 3′5'-dinitrosalicylic acid (DNS). J. Trop. Biol. 8, 359–363. doi: 10.15886/j.cnki.rdswxb.2017.03.018

[ref72] Zheng Y. J. Wang X. Y. Tian H. L. Li Y. Shi P. Q. Guo W. Y. . (2021). Effect of four modification methods on adsorption capacities and *in vitro* hypoglycemic properties of millet bran dietary fibre. Food Res. Int. 147:110565. doi: 10.1016/j.foodres.2021.110565, 34399541

[ref73] Zhou X. L. Duan M. J. Gao S. J. Wang T. Wang Y. B. Wang X. Y. . (2022). A strategy for reducing acrylamide content in wheat bread by combining acidification rate and prerequisite substance content of Lactobacillus and *Saccharomyces Cerevisiae*. Curr. Res. Food Sci. 5, 1054–1060. doi: 10.1016/j.crfs.2022.06.005, 35789803 PMC9249569

