# Correction: *Jiangshui*-derived lactic acid bacteria with high hypoglycemic potential: functional properties and fermentation performance of *Sonchus oleraceus Jiangshui*

**DOI:** 10.3389/fmicb.2026.1835597

**Published:** 2026-04-07

**Authors:** Xian-Gang Meng, Hui Yao, Qing-kai Jin, Tuan-Jie Che, Mohamed S. Sheteiwy, Afrah E. Mohammed, Modhi O. Alotaibi, Si-Jing Chang, Yan-Wen Gui

**Affiliations:** 1 Department of Bioengineering, School of Biological and Pharmaceutical Engineering, Lanzhou Jiaotong University, Lanzhou, China; 2 Innovation Center of Functional Genomics and Molecular Diagnostics Technology of Gansu Province, Engineering Laboratory for Biochips of Gansu Province, Lanzhou, China; 3 Department of Integrative Agriculture, College of Agriculture and Veterinary Medicine, United Arab Emirates University, Abu Dhabi, United Arab Emirates; 4 Department of Biology, College of Science, Princess Nourah Bint Abdulrahman University, Riyadh, Saudi Arabia; 5 Microbiology and Immunology Unit, Natural and Health Sciences Research Center, Princess Nourah Bint Abdulrahman University, Riyadh, Saudi Arabia

**Keywords:** lactic acid bacteria, fermentation, functional properties, *Sonchus oleraceus Jiangshui*, hypoglycemic activity, antibacterial properties

An incorrect number was provided in the **Funding** statement for Princess Nourah bint Abdulrahman University Researchers Support Project Number. The correct number is PNURSP2026R740.

The corrected **Funding** statement is below:

“The author(s) declared that financial support was received for this work and/or its publication. This work was funded by the Natural Science Foundation of Gansu Province (22JR5RA345), Science and Technology Program of Gansu Province (25CXGA034), and the Lanzhou Jiaotong University Youth Fund (1200060807). The authors are also thankful to Princess Nourah bint Abdulrahman University Researchers Supporting Project Number (PNURSP2026R740), Princess Nourah bint Abdulrahman University, Riyadh, Saudi Arabia, and the authors extend their appreciation to the financial support provided by the United Arab Emirates University, Abu Dhabi, United Arab Emirates, through the Startup Project Fund (Grant Code: G00005036) and Fund Code: 12F080.”

The original version of this article has been updated.

